# Supplementary material for: Subtype-resolved norepinephrine imaging with an engineered α1D-adrenergic receptor indicator
Source: Cell Rep Methods. 2026 Jul 22;6(9):101536. doi: 10.1016/j.crmeth.2026.101536 (PMC13615494; doi:10.1016/j.crmeth.2026.101536)
Supplement: Supplementary file 1 [file mmc1.pdf]

Cell Reports Methods, Volume *6*

Supplemental information

Subtype-resolved norepinephrine imaging with an engineered α_1D-adrenergic receptor indicator

Valentin Lu Rohner, Zacharoula Kagiampaki, Pauline Bohne, Antonia Renate Klein, Latife Sönmez, Laura Moreno Wasielewski, Musadiq A. Bhat, Andreas Reiner, Melanie D. Mark, and Tommaso Patriarchi

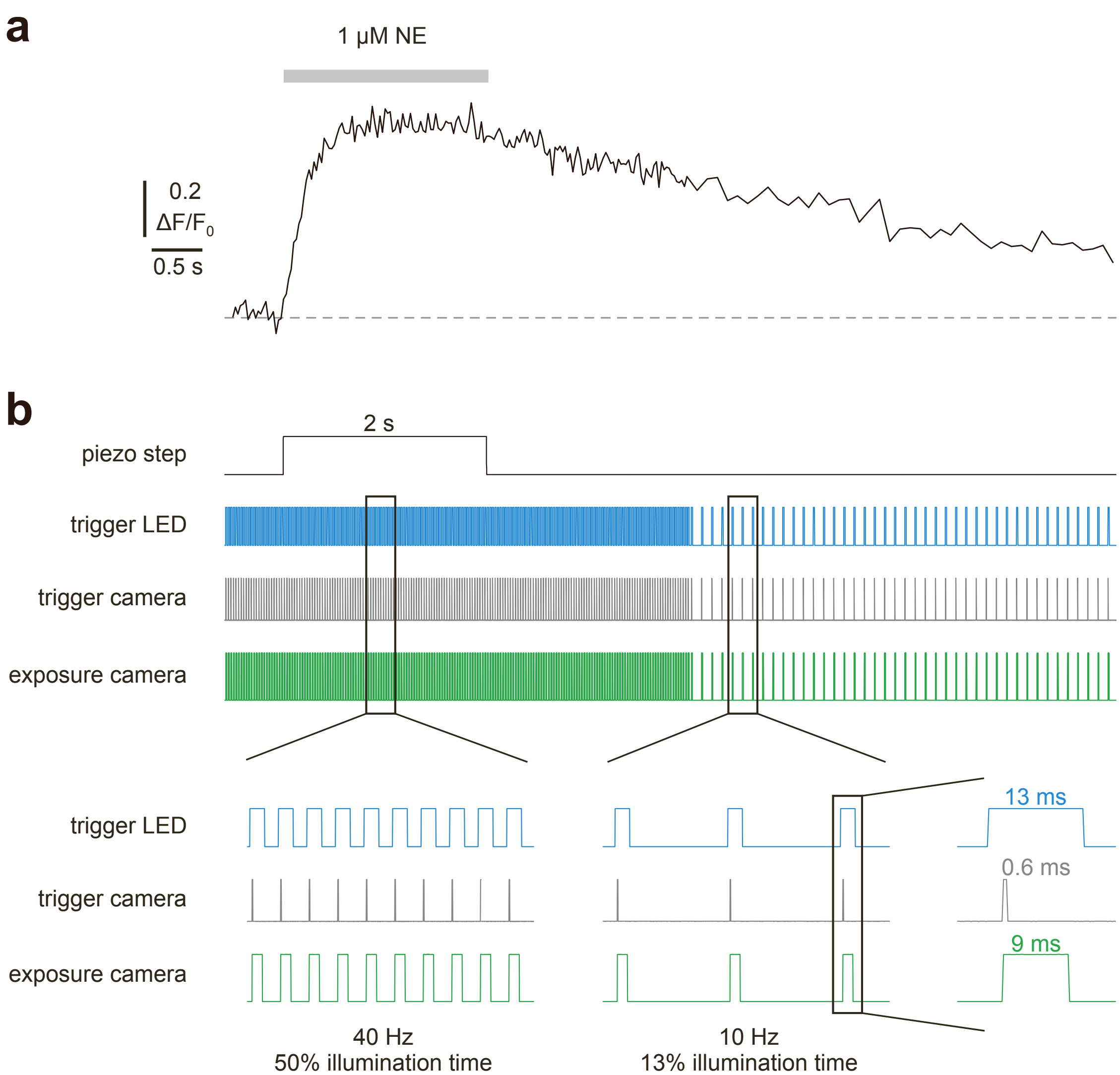

Supplementary Figure 1 | Patch-clamp fluorometry measurements with a split time-based protocol.

a, $\Delta F/F_0$ trace of Alpha1DLight (shortened from **Fig. 1h**). **b,** Trigger signals for the piezo element (solution exchange), 470 nm LED (illumination), camera acquisition (exposure start) and output signal denoting the actual camera exposure. As indicated, 2 s after the 2 s NE application the illumination and sampling episodes were reduced from 40 Hz to 10 Hz to achieve extended measurements times (14 - 20 s per sweep) with reduced photobleaching (illumination time reduced from 50% to 13%).

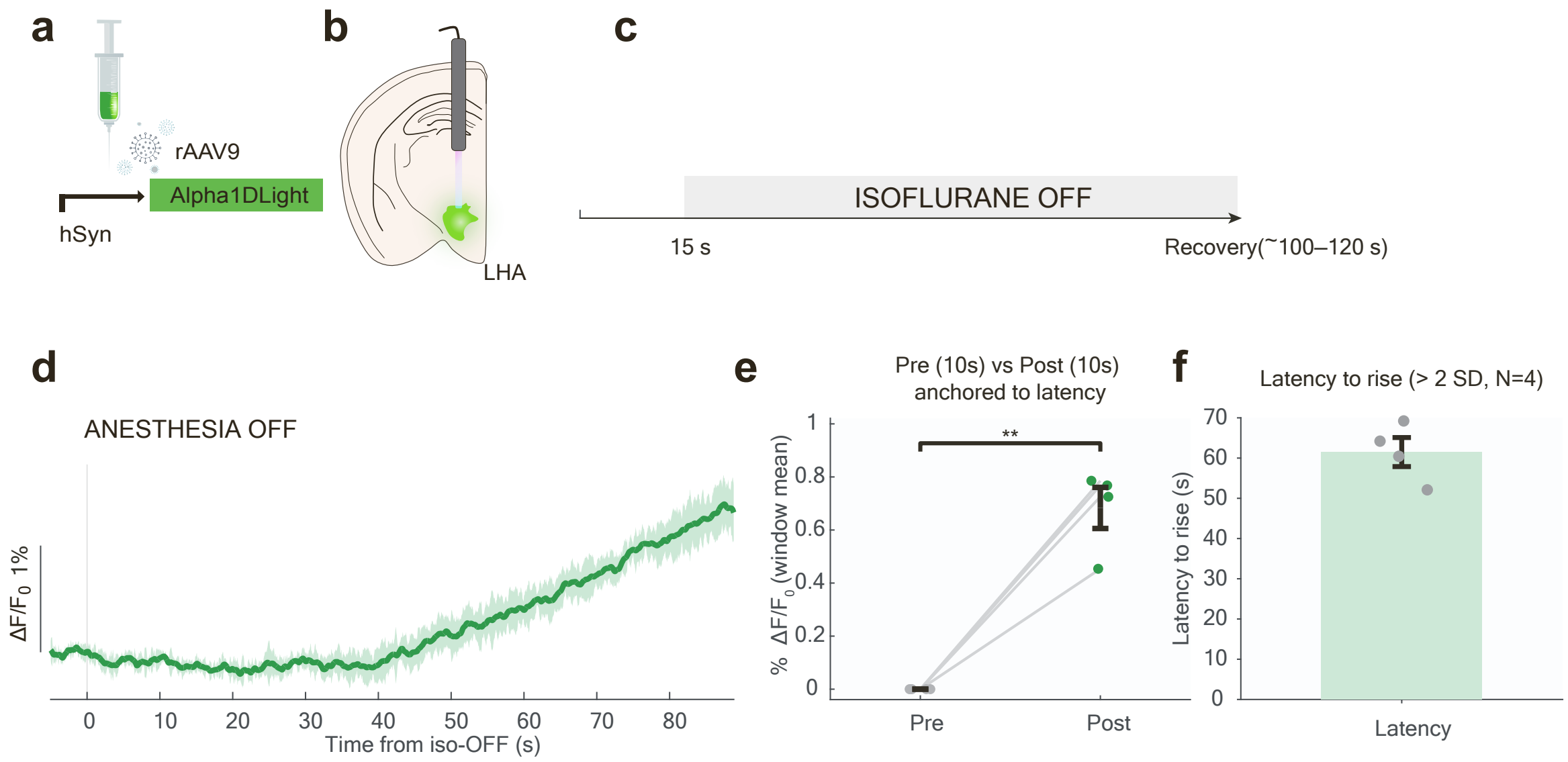

Supplementary Figure 2 | Alpha1DLight reports gradual NE increase upon recovery from isoflurane anesthesia

a-c, Schematic of Alpha1DLight expression in the lateral hypothalamus (LHA) and photometry recordings during isoflurane anesthesia till recovery. **d**, Averaged ΔF/F trace aligned to isoflurane offset (ISO-OFF), showing a gradual tonic increase in NE signal during recovery from anesthesia. **e**, Quantification of mean ΔF/F before isoflurane offset (−10 to 0 s relative to ISO-OFF) and after the onset of NE signal rise (0 to +10 s, relative to the detected latency for each mouse). NE levels were significantly increased following signal onset (two-tailed paired t-test, $t(3) = 8.82$, $p = 0.0031$; N = 4 mice). **f**, Latency to NE signal rise following ISO-OFF, defined as the time to exceed 2 standard deviations above baseline (N = 4 mice). Data are shown as mean ± SEM.
