# Supplementary material for: Subtype-resolved norepinephrine imaging with an engineered α1D-adrenergic receptor indicator
Source: Cell Rep Methods. 2026 Jul 22;6(9):101536. doi: 10.1016/j.crmeth.2026.101536 (PMC13615494; doi:10.1016/j.crmeth.2026.101536)
Supplement: Supplementary file 2 [file mmc3.pdf]

Cell Reports
Methods

Article

Subtype-resolved norepinephrine imaging with an engineered α_1D-adrenergic receptor indicator

Graphical abstract

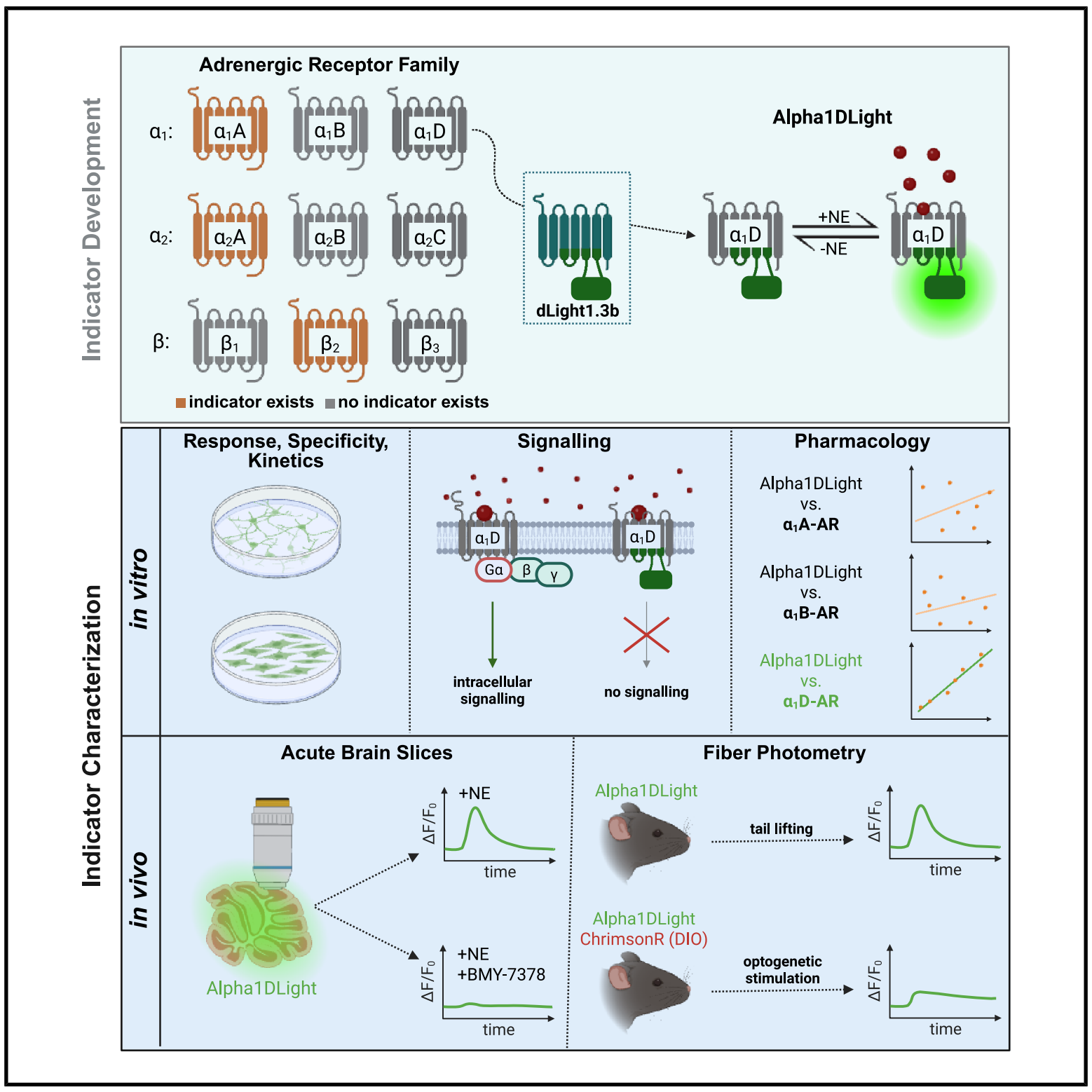

Highlights

- Engineering a human α1D indicator enables subtype-resolved norepinephrine imaging
- Enables optical detection of sustained norepinephrine signaling
- Direct comparison of α1A and α1D indicators reveals subtype-informed pharmacology
- We demonstrate detection of norepinephrine dynamics across systems

Authors

Valentin Lu Rohner, Zacharoula Kagiampaki, Pauline Bohne, ..., Andreas Reiner, Melanie D. Mark, Tommaso Patriarchi

Correspondence

patriarchi@pharma.uzh.ch

In brief

Rohner et al. develop Alpha1DLight, an α1D-adrenergic receptor-derived fluorescent indicator that enables subtype-resolved norepinephrine imaging. The sensor shows high affinity, reversible responses, and α1D-like pharmacological sensitivity and reports norepinephrine dynamics across cells, brain slices, and behaving mice, enabling investigation of sustained noradrenergic engagement and drug effects.

Rohner et al., 2026, Cell Reports Methods 6, 101536
September 21, 2026 © 2026 The Author(s). Published by Elsevier Inc.
https://doi.org/10.1016/j.crmeth.2026.101536

CellPress

Cell Reports Methods

CellPress
OPEN ACCESS

Article

Subtype-resolved norepinephrine imaging with an engineered α_1D-adrenergic receptor indicator

Valentin Lu Rohner,[1,5] Zacharoula Kagiampaki,[1,5] Pauline Bohne,[2] Antonia Renate Klein,[2] Latife Sönmez,[3] Laura Moreno Wasielewski,[3] Musadiq A. Bhat,[1] Andreas Reiner,[3] Melanie D. Mark,[2] and Tommaso Patriarchi[1,4,6,*]
[1]Institute of Pharmacology and Toxicology, University of Zürich, 8057 Zürich, Switzerland
[2]Behavioral Neuroscience, Ruhr University Bochum, 44801 Bochum, Germany
[3]Cellular Neurobiology, Department of Biology and Biotechnology, Ruhr University Bochum, 44801 Bochum, Germany
[4]Neuroscience Center Zurich, University and ETH Zürich, 8057 Zürich, Switzerland
[5]These authors contributed equally
[6]Lead contact
*Correspondence: patriarchi@pharma.uzh.ch
https://doi.org/10.1016/j.crmeth.2026.101536

MOTIVATION Norepinephrine acts through multiple adrenergic receptor subtypes whose distinct distributions and pharmacological properties are central to how noradrenergic signaling is interpreted across brain circuits. However, existing optical indicators sample only a narrow portion of this receptor landscape, limiting efforts to probe receptor subtype-resolved norepinephrine dynamics and drug action. Expanding indicator design to additional adrenergic receptor scaffolds could, therefore, provide not only new sensors but also new experimental access to the functional and pharmacological diversity of noradrenergic transmission.

SUMMARY

Genetically encoded fluorescent indicators enable optical measurement of neuromodulator dynamics, but available norepinephrine (NE) sensors draw from a limited subset of adrenergic receptor scaffolds. Here, we developed Alpha1DLight, an NE indicator engineered from the human α_1D-adrenergic receptor. In HEK293T cells, it showed large NE-evoked fluorescence responses ($\Delta F/F_0$ up to 612%), nanomolar apparent affinity for NE (EC_{50} = 76 nM), rapid activation (τ_{ON} = 193 ms), slow deactivation (τ_{OFF} = 6.75 s), and no coupling to downstream signaling. Pharmacological benchmarking against published α_1D-AR reference data indicated preservation of key α_1D-like ligand-response properties, supporting receptor-informed optical pharmacology. Alpha1DLight also reported reversible NE signals in cultured neurons, cerebellar slices, and behaving mice during fiber photometry, including endogenous release associated with arousal-related behavior and optogenetic activation of locus coeruleus projections. Together, these results establish an α_1D-derived, subtype-informed approach to NE imaging that complements existing sensors and is well suited for probing sustained noradrenergic engagement.

INTRODUCTION

Norepinephrine (NE) is a central neuromodulator that shapes arousal, attention, learning, motor output, and stress responsiveness.[1–5] These diverse functions arise from the heterogeneous distribution and sensitivities of adrenergic receptors (ARs) across brain regions and cell types, as well as their distinct signaling properties. The mammalian genome encodes nine adrenergic G protein-coupled receptors (GPCRs): three α_1 (α_1A, α_1B, α_1D), three α_2, and three β ARs, each with unique expression patterns in the central nervous system and G protein coupling profiles.[6,7] Understanding how individual receptor subtypes decode NE signals is essential for linking noradrenergic dynamics to cellular and behavioral outcomes and for developing more selective pharmacological interventions.

Genetically encoded fluorescent indicators (GEFIs) based on GPCR scaffolds have transformed the study of neuromodulation, enabling real-time measurements of extracellular neuromodulator dynamics with cell-type specificity and high spatiotemporal resolution.[8–11] Several NE indicators have been developed using α_1A-, α_2A-, or β_2-ARs as scaffolds, allowing imaging of NE release *in vitro* and *in vivo*.[12–15] However, the majority of AR subtypes remain unexploited as indicator frameworks, leaving significant gaps in the ability to study subtype-specific signaling, receptor pharmacology, or disease-relevant pathways involving poorly characterized receptors.

© 2026 The Author(s). Published by Elsevier Inc.
This is an open access article under the CC BY license (http://creativecommons.org/licenses/by/4.0/).

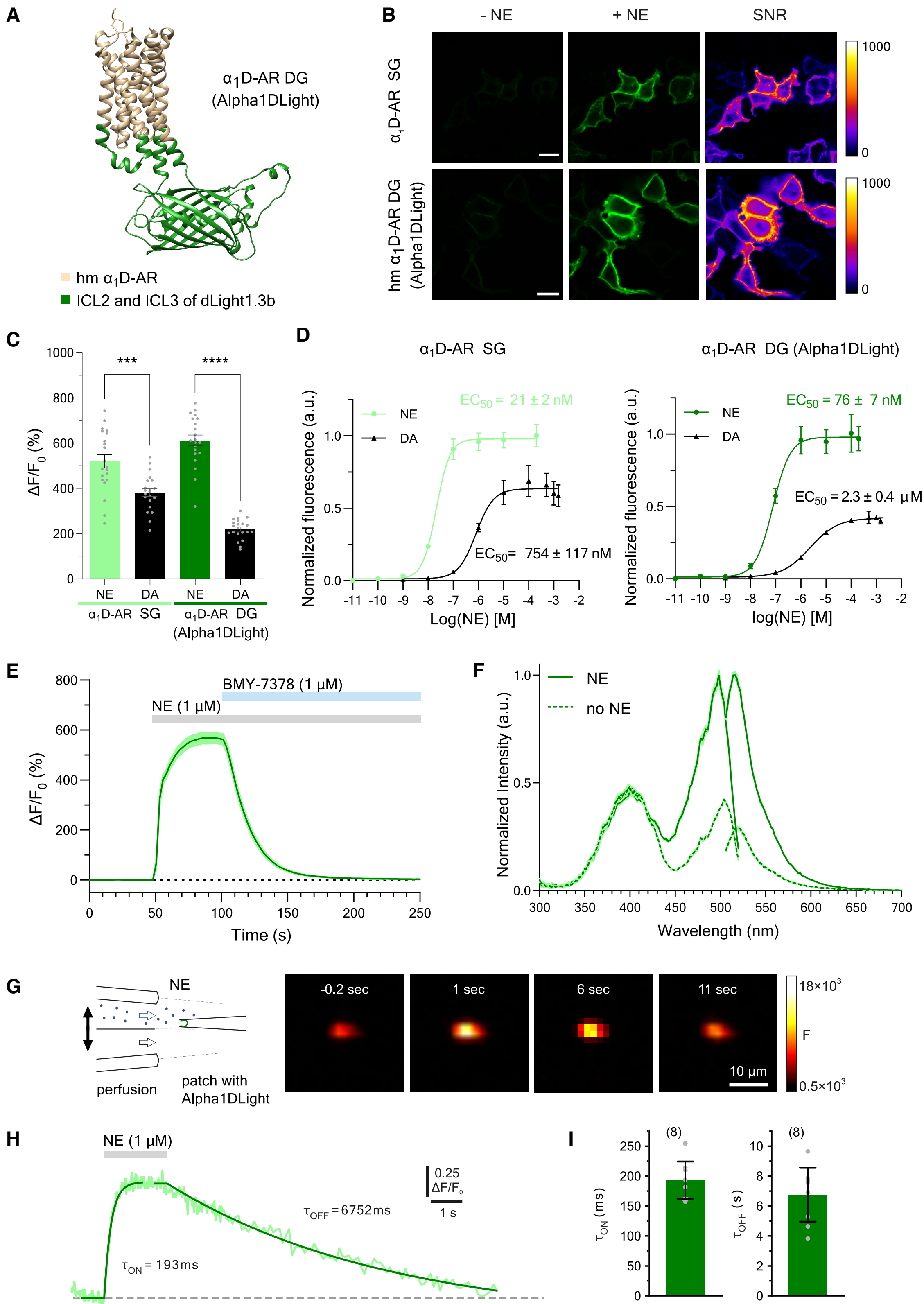

(legend on next page)

Among these, the α_1D-AR is of particular interest: it exhibits distinct expression in selected neuronal populations,[16,17] contributes to dendritic NE signaling, and has been implicated in stress-linked motor dysfunction and cerebellar pathophysiology.[18] Yet, no optical tools exist to monitor NE dynamics through this receptor. More broadly, the lack of a full receptor-subtype indicator panel limits opportunities to systematically probe ligand selectivity, receptor engagement, or more importantly, the functional roles of adrenergic diversity in neural circuits.

Here, we expand the adrenergic indicator family by engineering the first α_1D-AR-based fluorescent NE indicator, Alpha1DLight. Using a sequence-guided grafting strategy previously only tested on a small subset of GPCRs,[15] we transplanted a sensing module containing both the intracellular loop 2 and 3 (ICL2 and ICL3) domains of dLight1.3b[12] into the human α_1D-AR to generate a high-affinity indicator with a large dynamic range. We characterize its pharmacology and performance in HEK cells and primary neurons, demonstrate robust NE detection in acute cerebellar slices, and show that Alpha1DLight reports endogenous NE release *in vivo* during defined behavioral, pharmacological, and optogenetic paradigms. Rather than aiming to replace the existing NE indicators, Alpha1DLight is intended to complement them by enabling α_1D-like, receptor-subtype-resolved optical pharmacology, particularly in contexts where sustained noradrenergic engagement and subtype-dependent drug effects are of interest.

RESULTS

Development and characterization of α_1D-AR-based genetically encoded fluorescent indicators

We previously showed that the reporting module of dLight1.3b[12] can be transplanted onto a subset of other GPCRs using a Ballesteros-Weinstein (BW) alignment-guided grafting strategy.[15] Because this approach performed particularly well for α_1A-AR scaffolds, and given the structural and pharmacological similarities between adrenergic and dopaminergic receptors,[19,20] we applied the same strategy to engineer an indicator based on the human α_1D-AR.

We generated two indicator variants based on the human α_1D-AR by replacing the ICL3 alone (single graft [SG]; BW 5.63–6.33) or both ICL2 and ICL3 (double graft [DG]; BW 3.50–4.44 and 5.63–6.33) with the corresponding regions from dLight1.3b (Figure 1A). When transiently expressed in HEK293T cells, both indicator variants were fluorescent, were primarily located at the plasma membrane (Figure 1B), and responded with a large dynamic range of 519% ± 30% (α_1D AR SG, mean ± SEM) and 612% ± 24% (α_1D AR DG, mean ± SEM) to the application of 10 μM NE (Figure 1C).

Because NE and dopamine (DA) share high structural similarity and cross-activate each other's receptors,[22] it is essential to determine the selectivity of a new indicator among these two ligands. Application of a saturating concentration of DA (1 mM) onto HEK293T cells transiently expressing the indicators revealed that the efficacy of DA is 73.4% (α_1D-AR SG) and 35.9% (α_1D-AR DG) compared to that of NE (Figure 1C). Dose-response measurements in stable LLP-HEK cells[23] revealed nanomolar apparent affinity for NE (EC_{50}: 21 ± 2 nM for SG; 76 ± 7 nM for DG) and markedly reduced potency for DA (EC_{50}: 754 ± 117 nM for SG; 2.30 ± 0.43 μM for DG; Figure 1D). These values reflect a substantially (10- to 40-fold) higher apparent NE affinity of these indicators compared to previously reported α_1A-AR-based ones.[15] Based on its larger dynamic range and stronger NE-DA selectivity among the two indicator versions, the DG construct was selected for further characterization and was termed Alpha1DLight.

We next assessed the pharmacological reversibility of the indicator. Alpha1DLight-expressing HEK293T cells were stimulated with NE (1 μM) and subsequently treated with the α_1D-AR-selective antagonist BMY-7378 (1 μM). The antagonist was able to fully reverse the fluorescence increase to baseline (Figure 1E), confirming that the indicator can cycle between the active and inactive states. Excitation and emission spectra measured in cell suspensions showed maxima at 498 and 516 nm, respectively, and an isoemissive point for excitation at 416 nm (Figure 1F).

To examine the activation and deactivation kinetics of Alpha1DLight under precise ligand-delivery conditions, we performed patch-clamp fluorometry on HEK293T cells expressing

Figure 1. Engineering and *in vitro* characterization of α_1D-based norepinephrine indicators

(A) Structural model of Alpha1DLight generated using Alphafold2.[21] The N- and C-termini of the indicator are not shown. The original receptor as well as the LightG module are color coded according to the legend.

(B) Representative images of HEK293T cells expressing α_1D-AR SG or α_1D-AR DG (Alpha1DLight) before and after application of NE (10 μM). Corresponding pixel-wise signal-to-noise ratio (SNR) heatmaps are shown on the right. Scale bars, 20 μm.

(C) Quantification of maximal fluorescence response ($\Delta F/F_0$) for α_1D-AR SG and α_1D-AR DG expressed in HEK293T cells, upon application of NE (10 μM) or DA (1 mM). $n = 21$ cells from three independent experiments. Two-tailed Student's t test with Welch's correction. α_1D-AR SG NE vs. DA: $^{***}p = 3.20 \times 10^{-4}$; α_1D-AR DG NE vs. DA: $^{****}p = 2.07 \times 10^{-14}$. Data are mean ± SEM.

(D) Fluorescence intensity dose-response curves of α_1D-AR SG (left) and α_1D-AR DG (right) for NE and DA in HEK293T cells normalized to the maximum $\Delta F/F_0$ for each catecholamine. Datapoints were fitted with four-parameter dose-response curves. Mean EC_{50} values are shown. $n = 3$ wells from three independent experiments for each concentration. Data are mean ± SEM.

(E) Timelapse of fluorescence response ($\Delta F/F_0$) of Alpha1DLight in HEK293T cells upon application of NE (1 μM) at a 1:1 dilution factor followed by application of BMY-7378 (1 μM) at a 1:1 dilution factor. Data are mean ± SEM.

(F) One-photon fluorescence excitation (λ_{em} = 560 nm) and emission (λ_{ex} = 470 nm) spectra acquired from transiently transfected Alpha1DLight-expressing HEK293T cells in the presence (Sat) or absence (Apo) of NE (10 μM). Each trace is the average of three independent experiments. Data are mean ± SEM.

(G) Kinetic patch-clamp fluorometry measurements. At $t = 0$, NE was applied for 2 s to an outside-out patch by rapid movement of a double-barreled perfusion pipette (cartoon, left). Alpha1DLight fluorescence at the indicated time points (right).

(H) $\Delta F/F_0$ signal change (average of 4 NE applications to a single patch). ON and OFF kinetics follow single exponential kinetics (dark lines).

(I) Summary of time constants (mean ± SD, $n = 8$ patches). The OFF kinetics are ~32 times slower than the ON kinetics.

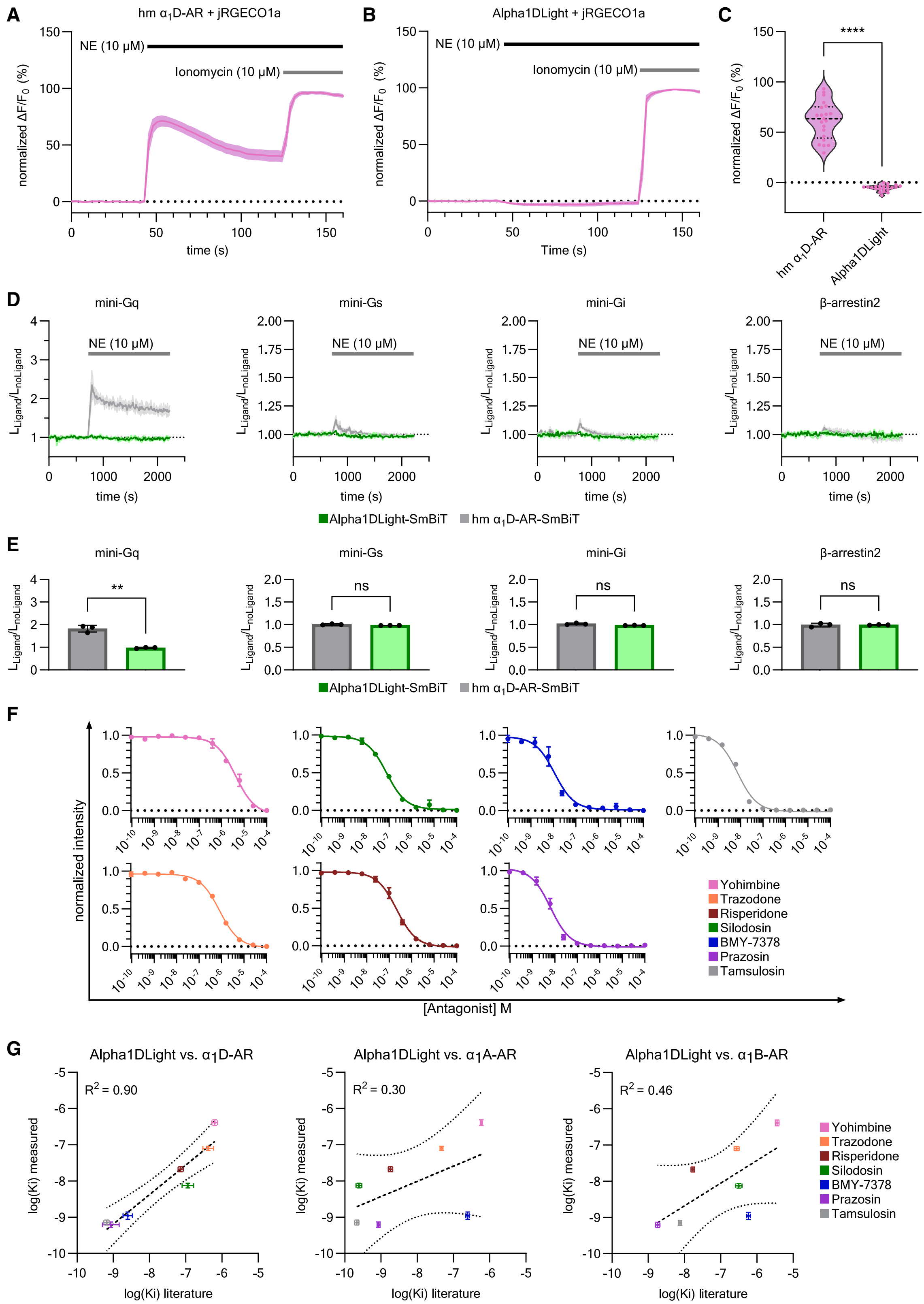

(legend on next page)

the sensor (Figure 1G; Figure S1). Rapid local application of 1 μM NE produced an immediate fluorescence increase followed by a gradual decay upon washout (Figure 1H). Analysis of the rising and falling phases revealed an activation time constant (τ_{ON}) of 193 ± 31 ms and a slow deactivation on the seconds timescale with a deactivation time constant (τ_{OFF}) of 6.75 ± 1.82 s (mean ± SD, n = 8 patches; Figure 1I; see also Table S1). These kinetic features are consistent with the high apparent affinity of Alpha1DLight and stand in clear contrast to the more rapid deactivation observed for previously reported medium-affinity α_1A-AR-based[15,24] or α_2A-AR-based[14] indicators. The relatively fast onset of Alpha1DLight demonstrates that ligand binding produces a rapid conformational change in the sensor, whereas the slow offset reflects prolonged ligand-receptor occupancy or gradual return to the basal state. Together, these measurements confirm that Alpha1DLight exhibits rapid activation but slow recovery, defining the temporal regime in which this α_1D-AR-based indicator most effectively reports NE fluctuations.

Intracellular signaling properties of Alpha1DLight

To determine whether grafting the dLight1.3b reporting module onto the α_1D-AR alters its native signaling capacity, we compared NE-evoked intracellular Ca^{2+} responses in cells expressing either the wild-type (wt) human α_1D-AR or Alpha1DLight, co-transfected with the red fluorescent Ca^{2+} indicator jRGECO1a.[25] Because NE triggers intracellular Ca^{2+} release through receptor-dependent signaling, whereas ionomycin directly increases cytosolic Ca^{2+} independently of receptor activation, we used ionomycin as a positive control for jRGECO1a function. Accordingly, activation of the wt α_1D-AR receptor produced a robust Ca^{2+} increase in response to 10 μM NE, followed by a strong ionomycin-evoked positive control (Figure 2A), whereas cells expressing Alpha1DLight showed no detectable NE-induced Ca^{2+} rise, despite maintaining a full ionomycin response (Figure 2B). Quantification of the responses confirmed a statistically significant difference between the strong Gq-linked Ca^{2+} signal of the wt receptor and the near-baseline response of Alpha1DLight (Figure 2C).

To more broadly assess downstream coupling, we performed nanoluciferase (NanoLuc) complementation assays using mini-G proteins representing the major GPCR signaling pathways (Gq, Gs, Gi) as well as β-arrestin2.[27,28] NE stimulation led to robust recruitment of mini-Gq in cells expressing the wt receptor, while no recruitment was detected in cells expressing Alpha1DLight (Figures 2D and 2E). In the same assay, neither the activation of the wt α_1D-AR nor Alpha1DLight led to substantial recruitment of mini-Gs, mini-Gi, or β-arrestin2, suggesting that this receptor subtype does not show strong preferential coupling to these intracellular signaling partners, at least under heterologous expression conditions (Figures 2D and 2E).

Together, these results demonstrate that Alpha1DLight behaves as a signaling-silent, ligand-responsive fluorescent indicator, similar to other GPCR-based GEFIs.[8,9,29]

Pharmacological characterization of Alpha1DLight and dual-color optical dissection of α_1-AR subtype pharmacology

To determine whether Alpha1DLight retained the pharmacological profile of human α_1D-AR, we measured the potency of seven small-molecule antagonists in stable LLP-HEK cells[23] expressing the indicator and compared the resulting values with published pharmacological data for the wt human α_1-AR subtypes.[26] Inhibition dose-response curves for yohimbine, trazodone, silodosin, risperidone, BMY-7378, prazosin, and tamsulosin were generated in the presence of 700 nM NE, corresponding to approximately 90% sensor saturation, and the IC_{50} values were determined (Figure 2F). Apparent K_i values were then calculated using the Cheng-Prusoff equation (see STAR Methods for details), substituting K_d with the experimentally determined EC_{50} of NE for Alpha1DLight. Comparison of these apparent K_i values with published reference values, obtained by [3H]-prazosin whole-cell competition binding in CHO cells,[26]

Figure 2. Signaling and pharmacological characterization of Alpha1DLight

(A) Normalized fluorescence response of the calcium indicator jRGECO1a co-expressed in HEK293T cells together with the wt hm α_1D-AR upon addition of NE (10 μM) and ionomycin (10 μM). n = 21 cells from three independent experiments. Data are mean ± SEM.

(B) Same as in (A) but for Alpha1DLight instead of the wt hm α_1D-AR. n = 21 cells from three independent experiments. Data are mean ± SEM.

(C) Statistical analysis of the time traces shown in (A and B). Data are shown as violin plot with the median (dashed black line) and quartiles (dotted black line). The mean normalized dynamic range of jRGECO1a upon addition of NE (10 μM) was compared using a two-tailed Student's t test with Welch's correction. ****p = 1.823×10^{-13}.

(D) Luminescence intensity ratio time traces of a nanoluciferase complementation assay to measure the recruitment of different mini-G proteins and β-arrestin2 by the wt hm α_1D-AR or Alpha1DLight upon addition of NE (10 μM). The experiment was performed in transiently transfected HEK293T cells co-expressing the hm α_1D-AR or Alpha1DLight with one of the three mini-G proteins or β-arrestin2. The wt receptor or indicator has a C-terminally fused natural peptide (NP), which can complement the LgBiT, which is N-terminally fused to the mini-G proteins or β-arrestin2, yielding a functional nanoluciferase.

Signals are shown as luminescence ratios normalized to control experiments where no ligand was added. Data are mean ± SEM.

(E) Statistical analysis of the time traces shown in (D). The mean normalized luminescence intensity ratio after addition of NE (10 μM) was compared between the condition with the hm α_1D-AR and Alpha1DLight using a two-tailed Student's t test with Welch's correction. mini-Gq: **p = 8.338×10^{-3}; mini-Gs: p = 0.108; mini-Gi: p = 0.079; β-arrestin2: p = 0.906. Data are mean ± SEM.

(F) Inhibition dose-response curves of Alpha1DLight for seven small-molecule antagonists measured in LLP-HEK cells in the presence of 700 nM norepinephrine (NE). Fluorescence signals were normalized to the maximal and minimal responses for each antagonist and fitted with four-parameter dose-response curves to determine the IC_{50} values. Data represent mean ± SEM of n = 3 independent experiments at each concentration.

(G) Correlation between log(K_i) values derived from Alpha1DLight measurements (F) and published[26] log(K_i) values for the wild-type human α_1D-AR (left), α_1B-AR (middle), and α_1A-AR (right). Solid lines indicate linear regression fits, with dotted lines representing the 95% confidence intervals. The R^2 of the linear regression and the Pearson's correlation coefficient was calculated for each receptor subtype (α_1D: R^2 = 0.90, r = 0.95, p = $0.105 \cdot 10^{-2}$; α_1A: R^2 = 0.30, r = 0.55, p = 0.199; α_1B: R^2 = 0.46, r = 0.68, p = 0.096). Data are shown as mean ± SEM.

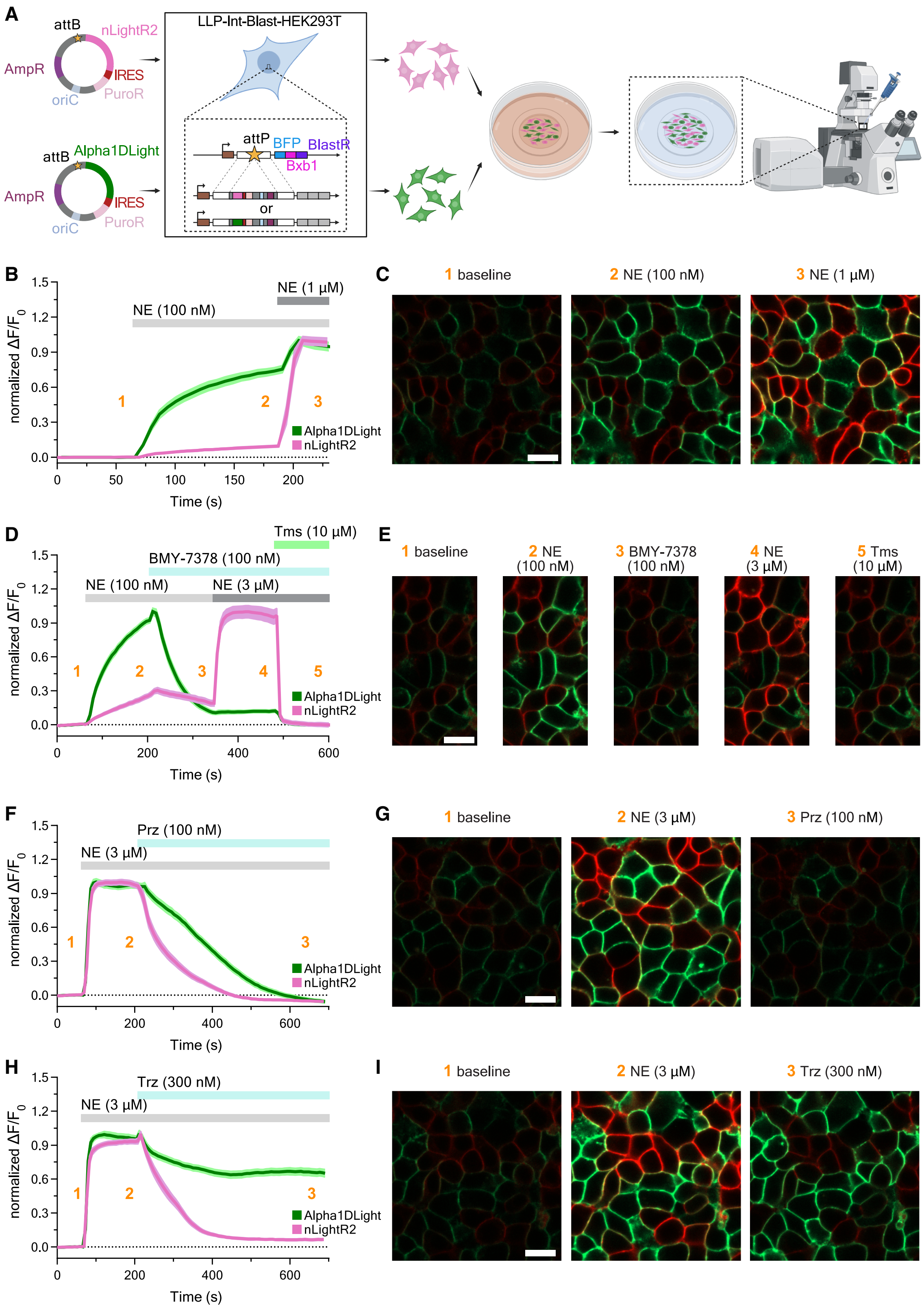

(legend on next page)

revealed the strongest correlation with the α_1D-AR subtype (Pearson's r = 0.95; *p* = 0.105 · 10^{-2}; *n* = 7), whereas correlations with the α_1A-AR subtype (Pearson's r = 0.55; *p* = 0.199; *n* = 7) and the α_1B-AR subtype (Pearson's r = 0.68; *p* = 0.096; *n* = 7) were substantially weaker (Figure 2G). Thus, Alpha1DLight preserves an α_1D-like pharmacological profile, supporting its use as a receptor-informed optical reporter for pharmacological measurements.

To further illustrate the practical utility of this subtype-resolved pharmacology, we performed dual-color imaging experiments directly comparing the green fluorescent α_1D-AR-based indicator Alpha1DLight to the red fluorescent α1A-AR-based indicator nLightR2.[15] Each sensor was stably expressed in a separate LLP-HEK cell line,[23] and both populations were imaged simultaneously within the same field of view using a dual-color acquisition strategy (Figure 3A).

A low NE concentration (100 nM) robustly activated Alpha1DLight, consistent with its high apparent affinity, whereas nLightR2 required a higher NE concentration (3 μM) to achieve comparable activation levels (Figures 3B and 3C). Next, we tested the effect of the α_1D-AR-selective antagonist BMY-7378 on Alpha1DLight and nLightR2. Activation of the α_1D-AR-based Alpha1DLight by 100 nM of NE was efficiently reversed by BMY-7378 (100 nM) and the response of the indicator to a subsequent addition of 3 μM NE was blocked, confirming that Alpha1DLight faithfully reports α_1D-like antagonist sensitivity (Figures 3D and 3E). In contrast, BMY-7378 did not block the activation of the α_1A-based nLightR2 upon addition of NE (3 μM). Tamsulosin (10 μM), a compound non-selective toward the α_1A-AR and α_1D-AR subtypes,[30] fully reversed the activation of both, Alpha1DLight and nLightR2 signals.

We next examined the effects of two clinically relevant α_1 adrenergic antagonists with distinct pharmacological profiles.[31–33] Prazosin (100 nM) attenuated both nLightR2 and Alpha1DLight signals to baseline levels, but with clearly distinct kinetics across the two indicators (Figures 3F and 3G). Whereas nLightR2 responses were rapidly suppressed, inhibition of Alpha1DLight was markedly slower, consistent with distinct ligand handling at the α_1D-derived indicator under these assay conditions (Figure 3F). In contrast, trazodone (300 nM) strongly suppressed α_1A-AR-based signals while producing weaker and incomplete suppression of Alpha1DLight responses under these conditions (Figures 3H and 3I). Across all conditions, the temporal sequence of ligand application and antagonist challenge produced reproducible, subtype-specific reversibility patterns that were internally consistent within each sensor but divergent across receptor backbones.

Validation of Alpha1DLight in cultured neurons and acute cerebellar slices

To assess neuronal performance, Alpha1DLight was expressed in primary rat cortical neurons under the control of a human synapsin promotor (hSyn) via recombinant adeno-associated virus (rAAV) transduction. The indicator showed robust membrane localization and clear fluorescence increases in response to 10 μM NE, whereas DA elicited substantially smaller signals. Furthermore, the responses could be fully reversed to baseline upon application of BMY-7378 (10 μM), mirroring the pharmacological profile observed in HEK293T cells (Figures 4A–4C). These findings confirm that Alpha1DLight preserves its dynamic range and ligand selectivity when expressed in neurons.

Given the strong noradrenergic innervation of the cerebellum and the known dendritic expression of α_1D-ARs in Purkinje cells (PCs),[18,34] we next evaluated Alpha1DLight in acute cerebellar slices. To achieve expression of the indicator in this cell type, an rAAV carrying Alpha1DLight driven by the cytomegalovirus (CMV) promotor was stereotactically injected into the cerebellar vermis of mice. Twelve to fifteen days post injection, acute sagittal brain slices were prepared and imaged using a confocal microscope. PCs displayed strong, dendritically enriched expression, enabling reliable fluorescence measurements (Figure 4D and 4E).

Bath application of 10 μM NE for 1 min produced a robust increase in $\Delta F/F_0$ of 52% ± 9%, peaking within ~3 min and followed by a gradual decrease toward baseline during washout ($\Delta F/F_0$ = 3% ± 2%) (Figure 4F). Quantification across slices confirmed a significant difference between peak NE responses and washout values (p = <0.0001), demonstrating effective sensor activation and reversibility *ex vivo*.

To assess repeated activation, slices were exposed to two sequential 10-μM NE pulses separated by a washout period (Figure 4G). Both applications triggered clear fluorescence

Figure 3. Dual-color optical dissection of α_1-AR engagement and pharmacology

(A) Schematic outline of the experimental procedure used to do dual-color imaging of Alpha1DLight and nLightR2 in HEK293T cells. For each indicator, a stably expressing HEK293T cell line was generated using a promoterless recombination vector encoding for the indicator and a puromycin resistance gene (PuroR). The recombination vector was used to transfect an LLP-HEK cell line with a stably integrated genomic landing pad under the control of a tetracycline-inducible promotor. One day prior to the experiment, the cell lines were mixed at a 1:1 ratio and seeded into 35-mm glass-bottom dishes. The experiment was conducted at on inverted confocal fluorescence microscope, and ligand solutions were manually applied using a micropipette. Created in https://BioRender.com.

(B) Time trace of the dynamic range ($\Delta F/F_0$) of Alpha1DLight (green) and nLightR2 (red) upon application of a low (100 nM, light gry bar) and high (3 μM, dark gray bar) concentration of NE, normalized to the maximal signal of each indicator. *n* = 21 cells from three independent experiments. Data are mean ± SEM.

(C) Representative images of the experiments analyzed in (B). The signal from Alpha1DLight is shown in green and the signal from nLightR2 in red. Images were generated by averaging the pixelwise intensity of the last five frames (30 s) prior to the next ligand application as marked with the orange numbers. Scale bar, 20 μm.

(D) Same as in (B) but for the addition of NE (100 nM, light gray bar), BMY-7378 (100 nM, light blue bar), NE (3 μM, dark gray bar), and tamsulosin (10 μM, light green bar).

(E) Same as in (C) but for the time trace shown in (D). Scale bar, 20 μm.

(F) Same as in (B and D) but for the addition of NE (3 μM) and prazosin (100 nM).

(G) Same as in (C and E) but for the time trace shown in (F). Scale bar, 20 μm.

(H) Same as in (B, D, and F) but for the addition of NE (3 μM) and trazodone (300 nM).

(I) Same as in (C, E, and G) but for the time trace shown in (H). Scale bar, 20 μm.

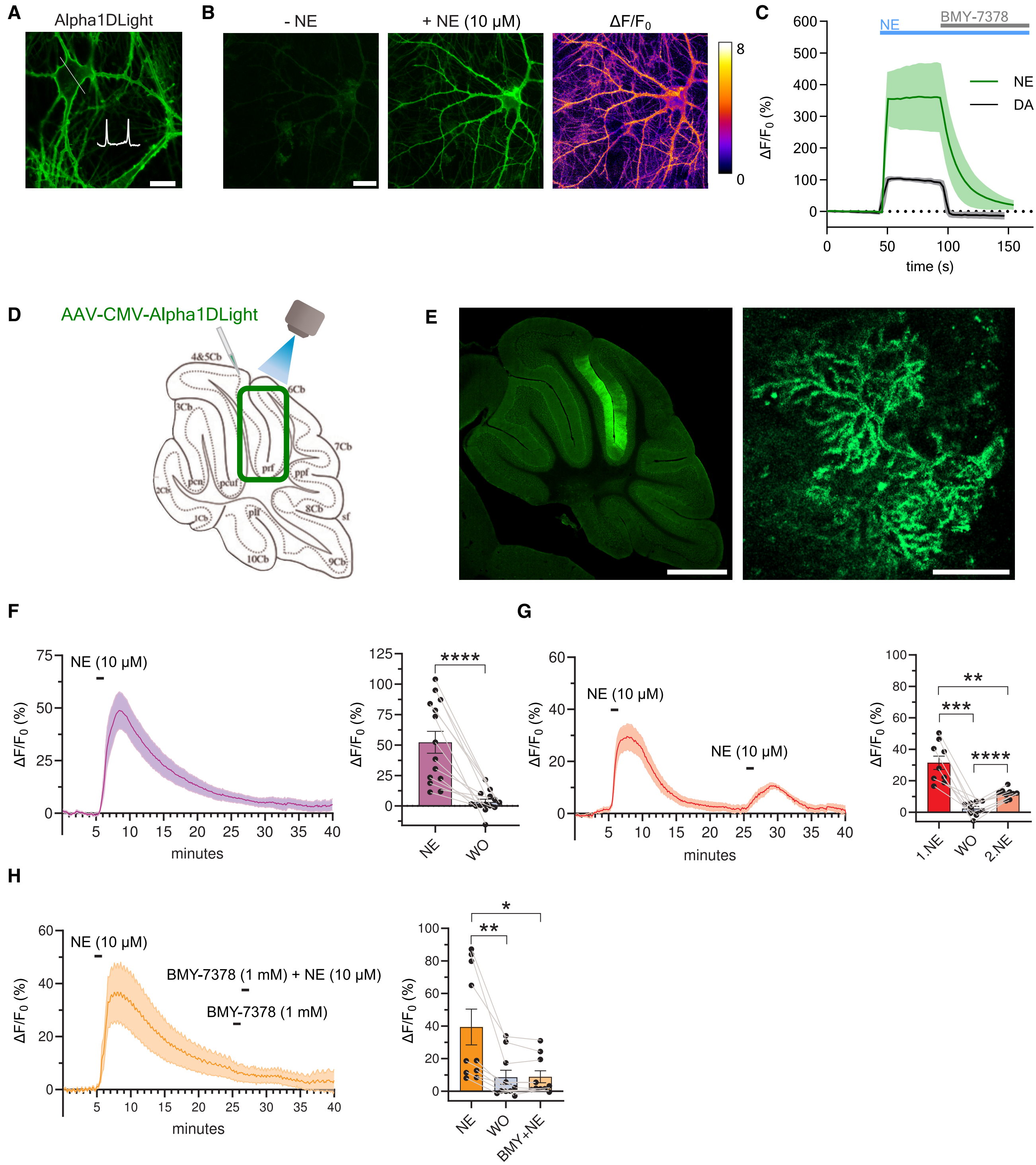

Figure 4. Robust Alpha1DLight responses in cultured neurons and cerebellar slices

(A) Representative image of primary cortical neurons expressing Alpha1DLight. Image was acquired in the presence of NE (1 μM). Fluorescence intensity plot along the white line is shown by the white inset. Scale bar, 20 μm.

(B) Representative images showing the fluorescence response of Alpha1DLight in primary cortical neurons upon application of NE (10 μM). Scale bar, 30 μm.

(C) Quantification of maximal fluorescence response ($\Delta F/F_0$) to bath application of NE or DA (10 μM) and subsequent BMY-7378 (10 μM). $n = 3$ regions of interest for NE and DA from three independent experiments. Data shown are mean ± SEM.

(legend continued on next page)

increases, although the second response was reduced in amplitude (first NE $\Delta F/F_0$ = 31.41% ± 4.239%; washout, WO, $\Delta F/F_0$ = 2.244% ± 1.461%; second NE $\Delta F/F_0$ = 11.86% ± 1.025%), possibly due to partial receptor desensitization during prolonged stimulation. The ability to evoke multiple responses indicates that Alpha1DLight remains functional across repeated activation cycles, with the reduced amplitude of the second response most likely reflecting photobleaching under these illumination conditions.

To test whether Alpha1DLight preserves α1D-like antagonist sensitivity *ex vivo* in PCs, we applied BMY-7378 (1 mM) before co-application with NE (10 μM). We found that the presence of BMY-7378 markedly suppressed NE-evoked fluorescence changes (NE $\Delta F/F_0$ = 39.38% ± 10.99%; WO $\Delta F/F_0$ = 8.528% ± 4.334%; BMY-7378+NE $\Delta F/F_0$ = 8.829% ± 3.647%; NE vs. WO p = 0.009; WO vs. BMY-7378 + NE p = 0.9796; NE vs. BMY-7378 + NE $^*p$ = 0.0122; Figure 4H), confirming that NE-induced increases in Alpha1DLight responses arise from ligand engagement at the binding pocket of the engineered α_1D-AR-based indicator rather than off-target effects. Together, these data establish that Alpha1DLight reports NE-dependent activation with strong, reversible signals in cerebellar PCs and preserves subtype-informed pharmacology in intact tissue.

Optical monitoring of NE release in awake behaving mice with Alpha1DLight

Given the robust NE-dependent activation and reversibility of Alpha1DLight observed in cultured neurons and acute cerebellar slices, we next asked whether the sensor could report endogenous NE release *in vivo* under physiological and controlled stimulation conditions. To this end, we expressed Alpha1DLight in the lateral hypothalamus ([LHA], a downstream target of locus coeruleus [LC] projections) and recorded fluorescence changes during behavioral arousal induced by tail-lift using fiber photometry (Figures 5A–5C). Each 60-s lift produced a rapid rise in $\Delta F/F_0$, followed by a sustained plateau throughout the suspension period and a slow decay after release, consistent with the slow off-kinetics expected from a high-affinity α_1D-AR-based sensor. Averaged traces across mice showed reliable, trial-to-trial responses (Figures 5D and 5E). Peak $\Delta F/F_0$ reached 4.67 ± 1.71% (mean ± SEM, n = 4), occurring ∼57 s after lift onset, with a large integrated response ($AUC_{0-60\ s}$ = 168.26% ± 68.21% $\Delta F/F_0$) and a prolonged decay ($\tau_{OFF} \approx 152$ s; Figures 5F and 5G). These features mirror the slow but sustained activation profile observed *ex vivo*.

To further characterize sensitivity to graded NE release under controlled conditions, we next combined fiber photometry with optogenetic stimulation of LC to LHA terminals (Figures 5H–5L). In anesthetized DBH-iCre mice[35] expressing ChrimsonR[36] in LC neurons, we delivered four stimulation protocols (5 Hz-1 s, 5 Hz-5 s, 5 Hz-10 s, 20 Hz-10 s), separated by long inter-trial intervals, while simultaneously recording Alpha1DLight signals (Figures 5H–5L). All stimulation paradigms evoked detectable increases in $\Delta F/F_0$ (Figure 5J). Consistent with the sensor's behavior *in vitro* and *ex vivo*, low-frequency stimulation (5 Hz) produced modest yet reproducible responses ($\Delta F/F_0$ = 0.14 ± 0.03 for 1 s, 0.18 ± 0.03 for 5 s, and 0.21 ± 0.03 for 10 s; mean ± SEM), whereas stronger activation (20 Hz-10 s) elicited substantially larger $\Delta F/F_0$ changes ($\Delta F/F_0$ = 0.81 ± 0.12; mean ± SEM), as visualized in trial-sorted heatmaps (Figure 5K). Across mice, peak NE responses differed significantly across stimulation protocols (repeated-measures ANOVA, $F(1.18, 3.53)$ = 40.22, p = 0.0045), with post hoc Tukey tests showing that the weakest protocol (5 Hz-1 s) produced significantly lower NE release than both 5 Hz-10 s (p = 0.0360) and 20 Hz-10 s (p = 0.0221) protocols and that the strongest protocol (20 Hz - 10 s) evoked significantly greater NE than both intermediate 5-Hz protocols (5 Hz-5 s, p = 0.0189; 5 Hz-10 s, p = 0.0197), while intermediate 5-Hz stimulation durations did not differ significantly from one another (Figure 5L). In addition to resolving arousal-evoked NE transients, we asked whether Alpha1DLight could also report slower, tonic changes in noradrenergic signaling associated with global brain state transitions. To this end, we monitored Alpha1DLight fluorescence during emergence from isoflurane anesthesia (Figures 2A–2C). Following discontinuation of anesthesia, Alpha1DLight signals increased gradually and reproducibly across animals, consistent with the progressive restoration of noradrenergic tone during the transition to wakefulness (Figures 2D and 2E).

Pharmacological modulation of arousal-evoked norepinephrine dynamics revealed by Alpha1DLight *in vivo*

Having established the ability of Alpha1DLight to report both evoked and tonic NE dynamics *in vivo*, we next used the arousal-evoked tail-lift paradigm as a controlled framework to

(D) Experimental schematic of *ex vivo* NE imaging in cerebellar Purkinje cells ([PCs], green box) expressing rAAV-CMV-Alpha1DLight following excitation with a blue laser at 488 nm.

(E) Representative images of Alpha1DLight expression in the cerebellar vermis (−6.5 mm AP) specifically in PCs, at 5× magnification (left; scale bar, 400 μM) and 40× magnification (right; scale bar, 20 μM).

(F) Rises in Alpha1DLight fluorescence were recorded after perfusion and subsequent washout (WO) of 10 μM NE (NE = 52.252% ± 8.984%; WO = 3.236% ± 2.288%; two-sided paired t test, $^{****}p \leq 0.0001$, n = 14 slices, n = 8 mice).

(G) Repetitive indicator activation with dual subsequent application of 10 μM NE starting after 5 min of baseline recording, with a WO period in between applications (left). (Right) Quantification of Alpha1DLight responses (1. NE = 31.41% ± 4.239%; WO = 2.244% ± 1.461%; 2. NE = 11.86% ± 1.025%; ordinary one-way ANOVA with multiple comparisons; 1. NE vs. WO: $^{***}p$ = 0.0001; WO vs. 2. NE: $^{****}p \leq 0.0001$; 1. NE vs. 2. NE: $^{**}p$ = 0.0021; n = 9 slices, n = 5 mice).

(H) Pre-incubation with the α_1D-AR antagonist BMY-7378 (1 mM, 1 min), followed by co-infusion of BMY-7378 and NE (10 μM, 1 min), abolished NE-evoked fluorescence increases in PCs (left). For comparison, 10 μM NE alone elicited a robust fluorescence response prior to BMY application. (Right) Quantification of Alpha1DLight responses across conditions (NE = 39.38% ± 10.99%; WO = 8.528% ± 4.334%; BMY-7378+NE = 8.829% ± 3.647%). Ordinary one-way ANOVA with multiple comparisons: NE vs. WO: $^{**}p$ = 0.009; WO vs. BMY-7378+NE n.s. (p = 0.9796); NE vs. BMY-7378+NE: $^*p$ = 0.0122. n = 10 slices, n = 6 mice. Data are mean ± SEM.

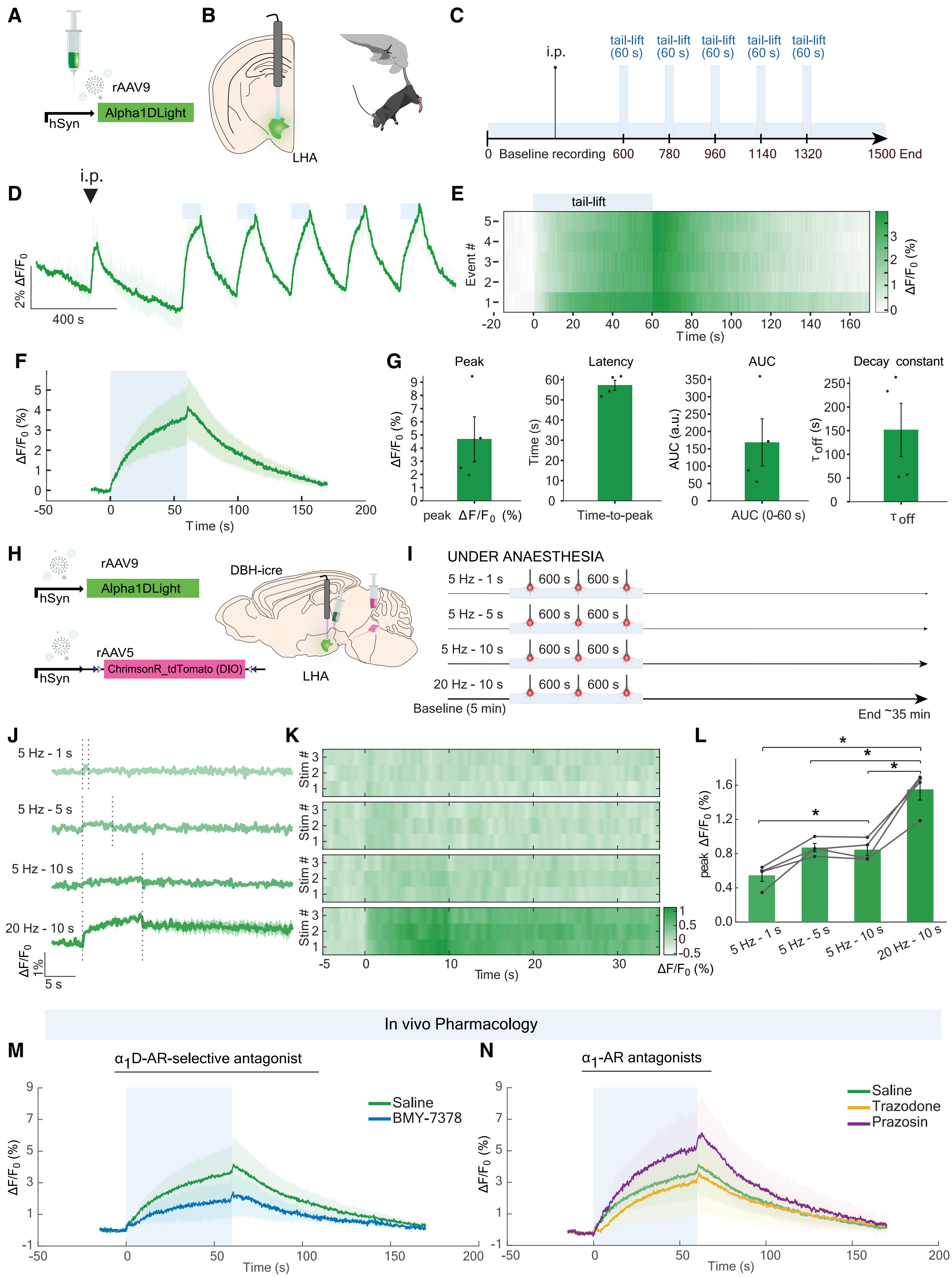

Figure 5. *In vivo* optical detection and pharmacological modulation of endogenous NE release with Alpha1DLight

(A and B) Schematic of viral strategy and recording configuration. Alpha1DLight was virally expressed in the lateral hypothalamus (LHA), and an optical fiber was implanted above the same region for photometry recordings.

(C) Experimental timeline for the tail-lift paradigm. Five 60-s tail-lift episodes were delivered with 180-s inter-trial intervals.

(D) Across-mice averaged full-session $\Delta F/F_0$ trace showing five lift-evoked responses.

(legend continued on next page)

examine how adrenergic pharmacology reshapes these responses. Tail-lift-aligned fluorescence responses were compared following systemic administration of the α_1D-selective antagonist BMY-7378 and clinically used,[37–39] nonselective α_1 antagonists with distinct pharmacological profiles. As a subtype-matched pharmacological probe, we first examined the effect of the α_1D-preferring antagonist BMY-7378. Under saline conditions, tail lifting consistently evoked a robust increase in Alpha1DLight $\Delta F/F_0$ (2.48 ± 1.00; mean ± SEM), whereas BMY-7378 (1 mg/kg) administration markedly attenuated tail-lift-evoked responses across events and animals ($\Delta F/F_0$ = 1.32 ± 0.78; mean ± SEM) (Figure 5M).

Having established sensitivity of Alpha1DLight to α_1D-selective antagonism during arousal, we next asked whether the sensor could be used to report how clinically used α_1-adrenergic blockers with distinct pharmacological profiles influence arousal-evoked NE dynamics *in vivo*. To address this, we examined the effects of trazodone and prazosin, which act broadly across α_1-AR subtypes and are employed in the clinics to treat depression and post-traumatic stress disorder, respectively.[38,40] In contrast to the marked suppression observed with the α_1D-selective antagonist, trazodone (10 mg/kg) had little effect on the amplitude or temporal profile of tail-lift-evoked Alpha1DLight responses ($\Delta F/F_0$ = 1.92 ± 1.34; mean ± SEM) (Figure 5N). This stands in clear distinction to its strong inhibitory effect on α_1A-based NE indicators previously reported by us[15] and is consistent with the α_1D-like pharmacological sensitivity of Alpha1DLight that we established *in vitro* (Figure 3).

Notably, the slow prazosin-induced suppression of Alpha1DLight observed in dual-color *in vitro* experiments (Figure 3F and 3G) predicts that α_1D-based signals may integrate competitive antagonism differently from α_1A-based indicators *in vivo*, particularly under conditions of sustained endogenous NE release. Prazosin administration (1 mg/kg) resulted in a modest enhancement of tail-lift-evoked Alpha1DLight responses ($\Delta F/F_0$ = 3.49 ± 1.35; mean ± SEM) (Figure 5N). This effect likely reflects systems-level modulation of NE dynamics, potentially through indirect peripheral effects or altered network feedback within arousal circuits.

Together, these observations suggest that Alpha1DLight can serve as an *in vivo* optical readout for distinguishing subtype-matched pharmacological effects from broader network-level consequences of clinically used α_1-adrenergic drugs.

DISCUSSION

In this study, we expand the repertoire of genetically encoded NE indicators by engineering Alpha1DLight, the first fluorescent sensor based on the human α_1D-AR. Using a sequence-guided grafting strategy, we transplanted the dLight1.3b reporting module into the ICL2 and ICL3 domains of α_1D-AR to generate a high-affinity, reversible indicator that preserves an α_1D-like pharmacological profile, as supported by antagonist profiling across multiple ligands and quantitative comparison with published α_1-AR subtype pharmacology.

A central advance of the present work is the pharmacological validation of Alpha1DLight against established α_1-AR subtype reference data. By profiling a panel of seven antagonists and comparing the resulting apparent K_i values with published pharmacology for the wt human α_1A-, α_1B-, and α_1D-ARs, we found the strongest correspondence with the α_1D subtype. Although this does not imply strict equivalence between the engineered sensor and the native receptor across all assay formats, it does show that our grafting procedure preserved the defining pharmacological features of α_1D-AR rather than collapsing subtype identity into a nonspecific adrenergic profile. In this sense, Alpha1DLight is best understood as an experimentally validated α_1D-like optical reporter rather than a merely receptor-inspired scaffold.

This pharmacological validation also provides the conceptual basis for both the dual-color *in vitro* experiments and the *in vivo* studies. Upon establishing the α_1D-like profile of the indicator, it became possible to deploy it alongside an α_1A-derived indicator to compare how well-characterized ligands are represented across distinct receptor-derived sensor backbones. Because the two sensors also operate over different effective concentration regimes, this dual-color configuration further enables simultaneous probing of ligand responses across a broader dynamic range than would be accessible with either sensor alone. The resulting experiments, therefore, serve as a proof of concept that receptor-diverse indicator panels can reveal ligand-dependent differences that would remain obscured in single-sensor assays.

(E) Per-event averaged heatmap of $\Delta F/F$ responses.
(F) Across-lifts and across-mice average $\Delta F/F_0$ trace aligned to lift onset (−15 to +170 s).
(G) Quantification of peak $\Delta F/F_0$, time to peak, AUC (0–60 s), and decay constant τ. Tail-lift evoked robust and sustained NE release across mice.
(H) Schematic of optogenetic stimulation of locus coeruleus (LC) to LHA terminals during Alpha1DLight photometry in anesthetized DBH-iCre mice.
(I) Experimental protocol showing four stimulation trains (5 Hz-1 s, 5 Hz-5 s, 5 Hz-10 s, 20 Hz-10 s), each separated by 600 s.
(J) Averaged $\Delta F/F_0$ traces for each stimulation protocol.
(K) Per-stimulation averaged heatmaps of $\Delta F/F_0$ responses across mice.
(L) Peak $\Delta F/F_0$ across stimulation frequencies and durations. Peak NE release differed significantly across protocols (repeated-measures ANOVA, $F(1.18, 3.53)$ = 40.22, *p = 0.0045). Tukey's post hoc tests revealed that the weakest protocol (5 Hz-1 s) evoked significantly lower NE release than both 5 Hz-10 s (*p = 0.0360) and 20 Hz-10 s (*p = 0.0221) protocols, while the strongest protocol (20 Hz-10 s) produced significantly greater NE release than both intermediate 5-Hz protocols (5 Hz-5 s, *p = 0.0189; 5 Hz-10 s, *p = 0.0197); intermediate 5-Hz stimulation durations did not significantly differ. n = 4 mice.
(M and N) *In vivo* pharmacological experiments. (M) Lift-aligned $\Delta F/F_0$ traces averaged across events and mice following systemic saline or the α_1D-AR-antagonist BMY-7378 (BMY) administration. Consistent with the α_1D-AR-based design of the sensor, BMY-7378 reduced tail-lift-evoked NE responses in the LHA relative to saline. (N) Lift-aligned $\Delta F/F_0$ traces averaged across events and mice during saline, trazodone, or prazosin administration, demonstrating drug-selective modulation of lift-evoked NE dynamics. Trazodone showed minimal impact, whereas prazosin enhanced and prolonged NE signals relative to saline. All data are shown as mean ± SEM.

Ex vivo experiments in cerebellar PCs confirmed the sensor's reliability and specificity in native tissue, while *in vivo* validation experiments showed that Alpha1DLight can report endogenous NE release from LC to LHA projections during behavioral arousal and optogenetic activation. The sensor produced gradual, sustained increases in $\Delta F/F_0$ that decayed slowly after stimulation. While the *in vivo* responses were modest in amplitude compared to other NE indicators,[14,15] they were consistent, stimulus-dependent, and graded across different levels of LC activation. This demonstrates that Alpha1DLight is well suited to monitor prolonged neuromodulatory states, complementing existing indicators that emphasize faster temporal resolution or lower affinity receptor scaffolds.

Importantly, the *in vivo* experiments extend the utility of Alpha1DLight beyond sensor validation and into the domain of systems neuropharmacology. By combining a reproducible arousal paradigm with subtype-informed optical readout, we show that an α_1D-based indicator can be used to interrogate how adrenergic drugs reshape endogenous NE dynamics in behaving animals. Thus, the primary contribution of Alpha1DLight is not superior kinetics or sensitivity, but optical access to NE binding through an α_1D-derived sensor with experimentally validated α_1D-like pharmacology.

A key insight emerging from the *in vivo* pharmacology is the divergent effect of clinically used α_1-adrenergic blockers on Alpha1DLight signals. Trazodone had minimal impact on arousal-evoked responses, despite its strong suppressive effect on α_1A-based NE indicators reported previously.[15] This contrast is consistent with the antagonist profiling and dual-color experiments, which together indicate that Alpha1DLight retains a pharmacological signature distinct from α_1A-based indicators. By contrast, prazosin produced a modest enhancement of arousal-evoked Alpha1DLight responses. Rather than reflecting simple antagonism of the α_1D-derived sensing module, this effect more likely arises from systems-level modulation of NE dynamics, for example, through altered network feedback, disinhibition within arousal circuits, or peripheral actions that secondarily influence central NE release. The fact that prazosin suppresses Alpha1DLight signals *in vitro*, yet enhances arousal-evoked signals *in vivo*, highlights the distinction between receptor-level pharmacology and the circuit-level consequences of systemic drug administration. Such effects would not be captured by receptor-centric *in vitro* assays, underscoring the value of *in vivo* optical readouts for resolving emergent pharmacological phenomena.

We do not propose Alpha1DLight as a universal replacement for existing NE indicators. Instead, its principal advantage is that it extends the current toolkit with the first α_1D-AR-based scaffold and enables α_1D-like optical pharmacology not accessible with α_1A-, α_2A-, or β_2-based indicators. Its high apparent affinity and slow off-kinetics make it less suitable for resolving brief phasic NE transients than faster indicators, but potentially advantageous for monitoring sustained NE elevations, comparing ligand behavior across receptor-derived indicators, and probing how drugs reshape α_1D-like receptor engagement over longer timescales. For guidance on sensor choice, we provide in Table S1 a side-by-side comparison of all currently available NE indicators, including scaffold, affinity/sensitivity, dynamic range, kinetics, selectivity, and recommended use cases.

More broadly, our findings illustrate how receptor subtype-specific indicators can enable pharmacological profiling of neuromodulatory systems *in vivo*. Drugs that are nominally classified by receptor affinity can exert distinct effects on endogenous transmitter dynamics depending on circuit context, state, and feedback architecture. By expanding the indicator repertoire to include α_1D-based indicators, Alpha1DLight enables experiments that disentangle receptor-level engagement from systems-level consequences, opening new opportunities for mechanistic neuropharmacology and for evaluating drug effects in physiologically relevant settings.

Beyond its immediate utility, this work also underscores the broader potential of the grafting strategy used here. The successful engineering of an α_1D-based sensor suggests that additional ARs (and potentially other neuromodulatory GPCRs) can be transformed into fluorescent indicators with predictable pharmacology and distinct affinity ranges. In future, constructing a complete panel of subtype-specific adrenergic indicators would open the door to comparative pharmacology, receptor-specific drug screening, and mechanistic dissection of noradrenergic circuit computations at unprecedented resolution.

In summary, Alpha1DLight adds a new dimension to the noradrenergic imaging toolkit by enabling optical readout of α_1D receptor engagement *in vitro*, *ex vivo*, and *in vivo*. Rather than replacing existing indicators, it expands the molecular diversity available for probing NE signaling, providing researchers with a receptor-specific tool that can illuminate the distinct contributions of α_1D-mediated neuromodulation across circuits and behavioral states.

Limitations of the study

An important limitation of this study is that Alpha1DLight was expressed using nonselective viral strategies rather than under endogenous α_1D-AR regulatory control. Thus, although our experiments support preservation of an α_1D-like pharmacological profile at the sensor level, they do not establish endogenous α_1D-restricted expression in the recorded cells. Future studies could address this by combining Alpha1DLight with knockin or bacterial artificial chromosome transgenic strategies[41] under endogenous α_1D-AR regulatory elements, or with α_1D- or cell type-specific Cre driver lines together with Cre-dependent viral delivery, to better align pharmacological subtype identity with endogenous cellular expression patterns.[42]

The present study also does not establish how Alpha1DLight performance may vary across brain regions, cell classes, or subcellular compartments with different NE clearance mechanisms, receptor environments, or expression levels. As a result, sensor behavior characterized in the lateral hypothalamus and cerebellar PCs may not fully generalize to other noradrenergic target regions.

Another limitation is that the *in vivo* characterization was performed in a restricted set of behavioral and stimulation paradigms. Although these experiments demonstrate sensitivity to endogenous and evoked NE release, they do not yet define the full range of physiological conditions over which Alpha1DLight can reliably report changes in noradrenergic tone.

CellPress
OPEN ACCESS

RESOURCE AVAILABILITY

Lead contact

Requests for further information, resources, and reagents should be directed to and will be fulfilled by the lead contact, Tommaso Patriarchi (patriarchi@pharma.uzh.ch).

Materials availability

- Plasmids for mammalian expression of Alpha1DLight have been deposited on Addgene (plasmid numbers: 254922–254923).
- AAV viruses are available through the Viral Vector Facility of the University and ETH Zürich (https://vvf.ethz.ch/).
- The Alpha1DLight stable cell line will be available upon request via an MTA with the UZH.

Data and code availability

- Data availability: The full sequence of Alpha1DLight has been deposited in GenBank (GenBank: PZ_232273)
- Code availability: Custom MATLAB scripts have been deposited on Zenodo (https://doi.org/10.5281/zenodo.20728770) and are available on GitHub at the following link: https://github.com/PatriarchiLab/Alpha1DLight
- Any additional information required to reanalyze the data reported in this work is available from the lead contact upon request.

ACKNOWLEDGMENTS

The results are part of a project that has received funding from the European Research Council (ERC) under the European Union's Horizon 2020 research and innovation program (grant agreement no. 891959 to T.P.). We also acknowledge funding from the Swiss National Science Foundation (project grant no. 320030E_224301 to T.P., 320030-236030 to T.P., and 310030L_212508 to T.P.) and the German Research Foundation (DFG, Deutsche Forschungsgemeinschaft; project grant nos. RTG 2862/1 to A.R., MA 5806/7-1 to M.D.M., MA 5806/1-2 to M.D.M., 316803389 [SFB1280] to M.D.M., and 492434978 [GRK2862/1] to M.D.M.). P.B. and M.D.M. were supported by the Ruhr University Bochum. We thank J.-C. Paterna and the Viral Vector Facility of the Neuroscience Center Zürich (ZNZ) for their help with virus production and Max Rybarski for helping set up the imaging in cerebellar slices. Some figure panels were created in https://BioRender.com. The graphical abstract was created in BioRender. Rohner, V. (2026) https://BioRender.com/iw7m5b2.

AUTHOR CONTRIBUTIONS

T.P. and V.L.R. conceived and T.P. led the study. V.L.R. performed all molecular cloning and *in vitro* indicator screening and characterization in HEK293T cells and neurons and analyzed data under the supervision of T.P. M.A.B. prepared primary cultures. L.S. and L.M.W. performed patch-clamp fluorometry experiments under the supervision of A.R. P.B. and A.R.K. performed imaging experiments in cerebellar slices under the supervision of M.D.M. Z.K. performed and analyzed *in vivo* photometry, optogenetic, and tail-lifting experiments and analyzed data under the supervision of T.P. All authors contributed to writing the manuscript.

DECLARATION OF INTERESTS

T.P. is a co-inventor on a patent application (PCT/US17/62993) related to the genetically encoded indicator technology described in this article.

DECLARATION OF GENERATIVE AI AND AI-ASSISTED TECHNOLOGIES IN THE WRITING PROCESS

During the preparation of this work, the authors used ChatGPT (OpenAI) to assist with language editing and clarity. After using this tool, the authors reviewed and edited the content as needed and take full responsibility for the content of the published article.

STAR★METHODS

Detailed methods are provided in the online version of this paper and include the following:

- KEY RESOURCES TABLE
- EXPERIMENTAL MODEL AND STUDY PARTICIPANT DETAILS
  - Cell lines
  - Animals
- METHOD DETAILS
  - Molecular cloning and viral production
  - Cell culture, confocal imaging and quantification
  - Patch-clamp fluorometry experiments
  - Plate-reader-based assays
  - Stereotactic surgeries
  - Acute brain slice preparation and imaging
  - Experiments with fiber photometry and optogenetics
  - Histology
- QUANTIFICATION AND STATISTICAL ANALYSIS
  - Statistical analysis

SUPPLEMENTAL INFORMATION

Supplemental information can be found online at https://doi.org/10.1016/j.crmeth.2026.101536.

Received: January 14, 2026
Revised: April 17, 2026
Accepted: June 26, 2026
Published: July 22, 2026

REFERENCES

1. Berridge, C.W. (2008). Noradrenergic modulation of arousal. Brain Res. Rev. *58*, 1–17. https://doi.org/10.1016/j.brainresrev.2007.10.013.
2. Berridge, C.W., and Waterhouse, B.D. (2003). The locus coeruleus–noradrenergic system: modulation of behavioral state and state-dependent cognitive processes. Brain Res. Rev. *42*, 33–84. https://doi.org/10.1016/S0165-0173(03)00143-7.
3. Sara, S.J. (2009). The locus coeruleus and noradrenergic modulation of cognition. Nat. Rev. Neurosci. *10*, 211–223. https://doi.org/10.1038/nrn2573.
4. Grueschow, M., Stenz, N., Thörn, H., Ehlert, U., Breckwoldt, J., Brodmann Maeder, M., Exadaktylos, A.K., Bingisser, R., Ruff, C.C., and Kleim, B. (2021). Real-world stress resilience is associated with the responsivity of the locus coeruleus. Nat. Commun. *12*, 2275. https://doi.org/10.1038/s41467-021-22509-1.
5. Burgess, C.R., and Peever, J.H. (2013). A noradrenergic mechanism functions to couple motor behavior with arousal state. Curr. Biol. *23*, 1719–1725. https://doi.org/10.1016/j.cub.2013.07.014.
6. Ahlquist, R.P. (1948). A study of the adrenotropic receptors. Am. J. Physiol. *153*, 586–600. https://doi.org/10.1152/ajplegacy.1948.153.3.586.
7. Hein, L. (2006). Adrenoceptors and signal transduction in neurons. Cell Tissue Res. *326*, 541–551. https://doi.org/10.1007/s00441-006-0285-2.
8. Wu, Z., Lin, D., and Li, Y. (2022). Pushing the frontiers: tools for monitoring neurotransmitters and neuromodulators. Nat. Rev. Neurosci. *23*, 257–274. https://doi.org/10.1038/s41583-022-00577-6.
9. Sabatini, B.L., and Tian, L. (2020). Imaging Neurotransmitter and Neuromodulator Dynamics In Vivo with Genetically Encoded Indicators. Neuron *108*, 17–32. https://doi.org/10.1016/j.neuron.2020.09.036.
10. Labouesse, M.A., and Patriarchi, T. (2021). A versatile GPCR toolkit to track in vivo neuromodulation: not a one-size-fits-all sensor. Neuropsychopharmacology *46*, 2043–2047. https://doi.org/10.1038/s41386-021-00982-y.

11. Patriarchi, T., Cho, J.R., Merten, K., Marley, A., Broussard, G.J., Liang, R., Williams, J., Nimmerjahn, A., von Zastrow, M., Gradinaru, V., and Tian, L. (2019). Imaging neuromodulators with high spatiotemporal resolution using genetically encoded indicators. Nat. Protoc. *14*, 3471–3505.

12. Patriarchi, T., Cho, J.R., Merten, K., Howe, M.W., Marley, A., Xiong, W.-H., Folk, R.W., Broussard, G.J., Liang, R., Jang, M.J., et al. (2018). Ultrafast neuronal imaging of dopamine dynamics with designed genetically encoded sensors. Science *360*, eaat4422. https://doi.org/10.1126/science.aat4422.

13. Oe, Y., Wang, X., Patriarchi, T., Konno, A., Ozawa, K., Yahagi, K., Hirai, H., Tsuboi, T., Kitaguchi, T., Tian, L., et al. (2020). Distinct temporal integration of noradrenaline signaling by astrocytic second messengers during vigilance. Nat. Commun. *11*, 471. https://doi.org/10.1038/s41467-020-14378-x.

14. Feng, J., Dong, H., Lischinsky, J.E., Zhou, J., Deng, F., Zhuang, C., Miao, X., Wang, H., Li, G., Cai, R., et al. (2024). Monitoring norepinephrine release in vivo using next-generation GRABNE sensors. Neuron *112*, 1930–1942.e6. https://doi.org/10.1016/j.neuron.2024.03.001.

15. Kagiampaki, Z., Rohner, V., Kiss, C., Curreli, S., Dieter, A., Wilhelm, M., Harada, M., Duss, S.N., Dernic, J., Bhat, M.A., et al. (2023). Sensitive multicolor indicators for monitoring norepinephrine in vivo. Nat. Methods *20*, 1426–1436. https://doi.org/10.1038/s41592-023-01959-z.

16. Day, H.E., Campeau, S., Watson, S.J., and Akil, H. (1997). Distribution of alpha 1a-alpha 1b- and alpha 1d-adrenergic receptor mRNA in the rat brain and spinal cord. J. Chem. Neuroanat. *13*, 115–139. https://doi.org/10.1016/s0891-0618(97)00042-2.

17. Schambra, U.B., Mackensen, G.B., Stafford-Smith, M., Haines, D.E., and Schwinn, D.A. (2005). Neuron specific alpha-adrenergic receptor expression in human cerebellum: implications for emerging cerebellar roles in neurologic disease. Neuroscience *135*, 507–523. https://doi.org/10.1016/j.neuroscience.2005.06.021.

18. Bohne, P., Josten, M., Rambuscheck, L., Brüggemann, J., Zhu, X., Rybarski, M.O., and Mark, M.D. (2025). Cerebellar α1D- adrenergic receptors mediate stress-induced dystonia in totteringtg/tg mice. Cell. Mol. Life Sci. *82*, 344. https://doi.org/10.1007/s00018-025-05843-1.

19. Xiao, P., Yan, W., Gou, L., Zhong, Y.-N., Kong, L., Wu, C., Wen, X., Yuan, Y., Cao, S., Qu, C., et al. (2021). Ligand recognition and allosteric regulation of DRD1-Gs signaling complexes. Cell *184*, 943–956.e18. https://doi.org/10.1016/j.cell.2021.01.028.

20. Toyoda, Y., Zhu, A., Kong, F., Shan, S., Zhao, J., Wang, N., Sun, X., Zhang, L., Yan, C., Kobilka, B.K., and Liu, X. (2023). Structural basis of α1A-adrenergic receptor activation and recognition by an extracellular nanobody. Nat. Commun. *14*, 3655. https://doi.org/10.1038/s41467-023-39310-x.

21. Mirdita, M., Schütze, K., Moriwaki, Y., Heo, L., Ovchinnikov, S., and Steinegger, M. (2022). ColabFold: making protein folding accessible to all. Nat. Methods *19*, 679–682. https://doi.org/10.1038/s41592-022-01488-1.

22. López, R.C., Noble, N., Özçete, Ö.D., Cai, X., Handy, G.E., Andersen, J.W., Patriarchi, T., Li, Y., and Kaeser, P.S. (2024). Innervation density governs crosstalk of GPCR-based norepinephrine and dopamine sensors. Preprint at bioRxiv. https://doi.org/10.1101/2024.11.23.624963.

23. Matreyek, K.A., Stephany, J.J., Chiasson, M.A., Hasle, N., and Fowler, D.M. (2019). An improved platform for functional assessment of large protein libraries in mammalian cells. Nucleic Acids Res. *48*, gkz910. https://doi.org/10.1093/nar/gkz910.

24. Rohner, V.L., Curreli, S., Lamothe-Molina, P.J., Kagiampaki, Z., Yee, A.G., Nardin, C., Eschholz, L., Dieter, A., Foustoukos, G., Milanese, P., et al. (2026). Next-generation multicolor indicators for in vivo imaging of norepinephrine. Nat. Methods *23*, 636–652. https://doi.org/10.1038/s41592-026-03006-z.

25. Dana, H., Mohar, B., Sun, Y., Narayan, S., Gordus, A., Hasseman, J.P., Tsegaye, G., Holt, G.T., Hu, A., Walpita, D., et al. (2016). Sensitive red protein calcium indicators for imaging neural activity. eLife *5*, e12727. https://doi.org/10.7554/eLife.12727.

26. Proudman, R.G.W., Pupo, A.S., and Baker, J.G. (2020). The affinity and selectivity of α-adrenoceptor antagonists, antidepressants, and antipsychotics for the human α1A, α1B, and α1D-adrenoceptors. Pharmacol. Res. Perspect. *8*, e00602. https://doi.org/10.1002/prp2.602.

27. Dixon, A.S., Schwinn, M.K., Hall, M.P., Zimmerman, K., Otto, P., Lubben, T.H., Butler, B.L., Binkowski, B.F., Machleidt, T., Kirkland, T.A., et al. (2016). NanoLuc Complementation Reporter Optimized for Accurate Measurement of Protein Interactions in Cells. ACS Chem. Biol. *11*, 400–408. https://doi.org/10.1021/acschembio.5b00753.

28. Nehmé, R., Carpenter, B., Singhal, A., Strege, A., Edwards, P.C., White, C.F., Du, H., Grisshammer, R., and Tate, C.G. (2017). Mini-G proteins: Novel tools for studying GPCRs in their active conformation. PLoS One *12*, e0175642. https://doi.org/10.1371/journal.pone.0175642.

29. Labouesse, M.A., Cola, R.B., and Patriarchi, T. (2020). GPCR-Based Dopamine Sensors-A Detailed Guide to Inform Sensor Choice for In vivo Imaging. Int. J. Mol. Sci. *21*, 8048. https://doi.org/10.3390/ijms21218048.

30. Richardson, C.D., Donatucci, C.F., Page, S.O., Wilson, K.H., and Schwinn, D.A. (1997). Pharmacology of tamsulosin: saturation-binding isotherms and competition analysis using cloned alpha 1-adrenergic receptor subtypes. Prostate *33*, 55–59. https://doi.org/10.1002/(sici)1097-0045(19970915)33:1%3C55::aid-pros9%3E3.0.co.

31. Basquez, R., and Pippin, M.M. (2025). Prazosin. In StatPearls (StatPearls Publishing).

32. Shin, J.J., and Saadabadi, A. (2025). Trazodone. In StatPearls (StatPearls Publishing).

33. Docherty, J.R. (2019). The pharmacology of α1-adrenoceptor subtypes. Eur. J. Pharmacol. *855*, 305–320. https://doi.org/10.1016/j.ejphar.2019.04.047.

34. Bloom, F.E., Hoffer, B.J., and Siggins, G.R. (1971). Studies on norepinephrine-containing afferents to Purkinje cells of rat cerebellum. I. Localization of the fibers and their synapses. Brain Res. *25*, 501–521. https://doi.org/10.1016/0006-8993(71)90457-4.

35. Parlato, R., Otto, C., Begus, Y., Stotz, S., and Schütz, G. (2007). Specific ablation of the transcription factor CREB in sympathetic neurons surprisingly protects against developmentally regulated apoptosis. Development *134*, 1663–1670. https://doi.org/10.1242/dev.02838.

36. Klapoetke, N.C., Murata, Y., Kim, S.S., Pulver, S.R., Birdsey-Benson, A., Cho, Y.K., Morimoto, T.K., Chuong, A.S., Carpenter, E.J., Tian, Z., et al. (2014). Independent optical excitation of distinct neural populations. Nat. Methods *11*, 338–346. https://doi.org/10.1038/nmeth.2836.

37. Kung, S., Espinel, Z., and Lapid, M.I. (2012). Treatment of nightmares with prazosin: a systematic review. Mayo Clin. Proc. *87*, 890–900. https://doi.org/10.1016/j.mayocp.2012.05.015.

38. Reist, C., Streja, E., Tang, C.C., Shapiro, B., Mintz, J., and Hollifield, M. (2021). Prazosin for treatment of post-traumatic stress disorder: a systematic review and meta-analysis. CNS Spectr. *26*, 338–344. https://doi.org/10.1017/S1092852920001121.

39. Coin, A., Malara, A., Noale, M., Trevisan, C., Devita, M., Abbatecola, A.M., Gareri, P., Del Signore, S., Bellelli, G., Fumagalli, S., et al. (2024). Real-World Use of Trazodone in Older Persons in Long Term Care Setting: A Retrospective Study. Int. J. Geriatr. Psychiatr. *39*, e70009. https://doi.org/10.1002/gps.70009.

40. Fagiolini, A., González-Pinto, A., Miskowiak, K.W., Morgado, P., Young, A.H., and Vieta, E. (2023). Role of trazodone in treatment of major depressive disorder: an update. Ann. Gen. Psychiatry *22*, 32. https://doi.org/10.1186/s12991-023-00465-y.

41. Gong, S., Zheng, C., Doughty, M.L., Losos, K., Didkovsky, N., Schambra, U.B., Nowak, N.J., Joyner, A., Leblanc, G., Hatten, M.E., and Heintz, N. (2003). A gene expression atlas of the central nervous system based on bacterial artificial chromosomes. Nature *425*, 917–925. https://doi.org/10.1038/nature02033.

42. Sjulson, L., Cassataro, D., DasGupta, S., and Miesenböck, G. (2016). Cell-Specific Targeting of Genetically Encoded Tools for Neuroscience. Annu.

Rev. Genet. *50*, 571–594. https://doi.org/10.1146/annurev-genet-120215-035011.

43. Laschet, C., Dupuis, N., and Hanson, J. (2019). A dynamic and screening-compatible nanoluciferase-based complementation assay enables profiling of individual GPCR-G protein interactions. J. Biol. Chem. *294*, 4079–4090. https://doi.org/10.1074/jbc.RA118.006231.

44. Schindelin, J., Arganda-Carreras, I., Frise, E., Kaynig, V., Longair, M., Pietzsch, T., Preibisch, S., Rueden, C., Saalfeld, S., Schmid, B., et al. (2012). Fiji: an open-source platform for biological-image analysis. Nat. Methods *9*, 676–682. https://doi.org/10.1038/nmeth.2019.

45. Edelstein, A.D., Tsuchida, M.A., Amodaj, N., Pinkard, H., Vale, R.D., and Stuurman, N. (2014). Advanced methods of microscope control using μManager software. J. Biol. Methods *1*, e10. https://doi.org/10.14440/jbm.2014.36.

46. Kubitschke, M., Müller, M., Wallhorn, L., Pulin, M., Mittag, M., Pollok, S., Ziebarth, T., Bremshey, S., Gerdey, J., Claussen, K.C., et al. (2022). Next generation genetically encoded fluorescent sensors for serotonin. Nat. Commun. *13*, 7525. https://doi.org/10.1038/s41467-022-35200-w.

47. Pollok, S., and Reiner, A. (2020). Subunit-selective iGluR antagonists can potentiate heteromeric receptor responses by blocking desensitization. Proc. Natl. Acad. Sci. USA *117*, 25851–25858. https://doi.org/10.1073/pnas.2007471117.

48. Wan, Q., Okashah, N., Inoue, A., Nehmé, R., Carpenter, B., Tate, C.G., and Lambert, N.A. (2018). Mini G protein probes for active G protein–coupled receptors (GPCRs) in live cells. J. Biol. Chem. *293*, 7466–7473. https://doi.org/10.1074/jbc.RA118.001975.

49. Sanders, J.I., and Kepecs, A. (2014). A low-cost programmable pulse generator for physiology and behavior. Front. Neuroeng. *7*, 43. https://doi.org/10.3389/fneng.2014.00043.

STAR★METHODS

KEY RESOURCES TABLE

REAGENT or RESOURCE	SOURCE	IDENTIFIER
Bacterial and virus strains		
NEB® 10-beta Competent *E. coli*	New England Biolabs	Cat#C3019H
NEB® Stable Competent E. coli	New England Biolabs	Cat#C3040H
AAV9-CMV-Alpgha1DLight-WPRE	Viral Vector Facility – University of Zurich	N/A
AAV9-hSyn1-Alpha1DLight-WPRE	Viral Vector Facility – University of Zurich	N/A
AAV5-hSyn-FLEX-ChrimsonR-tdTomato	UNC Vector Core	N/A
Chemicals, peptides, and recombinant proteins		
BMY 7378 dihydrochloride	hellobio	Cat#B134
BMY 7378 dihydrochloride	MedChemExpress	Cat#HY-100554
Laminin	Sigma-Aldrich	Cat#L2020
L-Norepinephrine-hydrochloride	Sigma-Aldrich	Cat#74480
Prazosin hydrochloride	CAYMAN	Cat#15023
Poly-D-lysine hydrobromide	Sigma-Aldrich	Cat#P6407
Polyethylenimine, branched, MW ~25,000	Sigma-Aldrich	Cat#408727
Risperidone	TargetMol	Cat#T0351
Silodosin	TargetMol	Cat#T1504
Tamsulosin hydrochloride	Tokyo Chemical Industry	Cat#T2749
Trazodone hydrochloride	Sigma-Aldrich	Cat#T6154
Yohimbine hydrochloride	Sigma-Aldrich	Cat#Y3125
Blasticidin	InvivoGen	Cat#ant-bl-05
Puromycin	InvivoGen	Cat#ant-pr-1
Doxycycline hyclate	Sigma-Aldrich	Cat#D9891
Critical commercial assays		
Nano-Glo® Live Cell Assay System	Promega	Cat#N2011
Deposited data		
Custom MATLAB scripts used for photometry data analysis	This paper	https://doi.org/10.5281/zenodo.20728770
Custom MATLAB scripts used for photometry data analysis	This paper	https://github.com/PatriarchiLab/Alpha1DLight
Experimental models: Cell lines		
HEK293tsa	in-house culture, (Cellular Neurobiology, RUB)	N/A
HEK293T	ATCC	Cat#CRL-3216; RRID: CVCL_0063
LLP-Int-Blast-HEK293T	Generated and obtained from Prof. Douglas M. Fowler; Matreyek et al. 2020	N/A
Experimental models: Organisms/strains		
C57BL/6J mouse (JAX^{TM})	Jackson Laboratory	Ca#000664; RRID: IMSR_JAX:000664
Heterozygous C57BL/6-Tg(Dbh-iCre)1Gsc (Dbh-iCre)	Generated by Prof. Günther Schütz; obtained from an in-house breeding colony	N/A
Recombinant DNA		
pCMV_hmα1DAR	this paper	N/A
pCMV_α1DAR_SG	this paper	N/A
pCMV_Alpha1DLight (α1DAR_DG)	this paper	Addgene Plasmid: 254922
attB_α1DAR_SG	this paper	N/A
attB_Alpha1DLight	this paper	N/A

(*Continued on next page*)

Continued

REAGENT or RESOURCE	SOURCE	IDENTIFIER
pAAV_hSyn_Alpha1DLight	this paper	Addgene Plasmid: 254923
pAAV_CMV_Alpha1DLight	this paper	N/A
pGP_CMV_NES_jRGECO1a	Dana et al.[25]	Addgene Plasmid: 61563
pCMV_LgBiT-miniGsq	Kagiampaki et al.[15]	N/A
pCMV_LgBiT-miniGsi	Kagiampaki et al.[15]	N/A
pCMV_LgBiT-miniGs	Kagiampaki et al.[15]	N/A
pNBe3_LgBiT-βarrestin2	Laschet et al.[43]	N/A
pCMV_hmα1DAR-SmBiT	this paper	N/A
pCMV_Alpha1DLight-SmBiT	this paper	N/A
Software and algorithms		
Fiji	Open-Source Community	https://fiji.sc/
Graphpad Prism 8.2.1	Graphpad Software Inc., Boston, USA	Prism 8.2.1 Release Notes
MATLAB	MathWorks Inc., Natick, MA, USA	R2025b
Doric Neuroscience Studio	Doric Lensed Inc., Quebec, Canada	https://neuro.doriclenses.com/products/doric-neuroscience-studio
Clampfit 11.1	Molecular Devices	https://www.moleculardevices.com
ImageJ 1.54f72	Schindelin et al.[44]	https://imagej.net
Micro-Manager 2.0b	Edelstein et al.[45]	https://micro-manager.org
OriginPro 2023	OriginLab Corporation	https://www.originlab.com
pClamp 10.7	Molecular Devices	https://www.moleculardevices.com
ProFit 7.1	Quantumsoft	https://quansoft.com
ZEN Microscopy Software	Zeiss	https://www.zeiss.com/microscopy/en/products/software/zeiss-zen-lite.html
Other		
Pulse Pal pulse train generator, Gen 2	Sanworks, Rochester, NY, USA	Product ID: 1102

EXPERIMENTAL MODEL AND STUDY PARTICIPANT DETAILS

Cell lines

HEK293T cells (CRL-3216; ATCC) were authenticated by the supplier and cultured at 37°C and 5%vol CO_2 in DMEM (41966–029; ThermoFisher) supplemented with 10%vol of FBS (A5256701; ThermoFisher) and Anti Anti (15240062; ThermoFisher).

To generate isogenic stable cell lines expressing the indicators, LLP-Int-Blast-HEK293T cells (LLP HEK)[23] were used. Prior to recombination, LLP-HEK cells were maintained like wild type HEK293T cells but in media supplemented with Doxycycline hyclate (2.5 μg/mL) and Blasticidin (20 μg/mL). The recombined cells were maintained in LLP-HEK cells like wild type HEK293T cells but in media supplemented with Doxycycline hyclate (2.5 μg/mL) and Puromycin (1 μg/mL).

Animals

All animal procedures complied with national and institutional regulations for the care and use of laboratory animals. Cerebellar NE imaging experiments were performed in adult C57BL/6 mice of both sexes (6–32 weeks old, 7 females, 6 males), housed individually under a 12 h light/dark cycle with *ad libitum* access to food and water. Procedures were conducted in accordance with the European Communities Council Directive 2010/63/EU and approved by the Bezirksamt Arnsberg and the Animal Care Committee of North Rhine-Westphalia (LAVES, Recklinghausen, Germany), with oversight from the Animal Welfare Commission of the Ruhr-University Bochum.

In vivo photometry and optogenetic experiments were carried out in heterozygous DBH-iCre mice (C57BL/6 background; males; >6 weeks old at the time of surgery)[35] These experiments adhered to the European Community Council Directive and the Swiss Animal Welfare Ordinance (TSchV 455.1), with approval from the Zurich Cantonal Veterinary Office. Mice were maintained in temperature (21°C–24°C)- and humidity (40–60%)-controlled conditions on a 12 h light–dark cycle with *ad libitum* access to food and water, and were group-housed. All surgical and behavioral procedures were performed during the light phase. Every effort was made to minimize the number of animals used and to reduce discomfort throughout the study.

METHOD DETAILS

Molecular cloning and viral production

The gene encoding for the human α_1D-AR was codon optimized, ordered as a gene block and inserted into a pCMV backbone (#217656, Addgene) with an N-terminally fused HA-secretory sequence and FLAG tag (pCMV_hmα1DAR). To clone the two indicator variants (α_1D-AR SG: pCMV_α1DAR_SG and the α_1D-AR DG (Alpha1DLight): pCMV_Alpha1DLight (α1DAR_DG)) the ICL2 region (BW numbering: 3.50–4.44) and/or ICL3 region (BW numbering: 5.63–6.33)) of the human α_1D-AR were replaced with the respective regions of dLight1.3b by PCR amplification (PfuUltra II Hotstart; Agilent) and Gibson assembly (NEBuilder DNA HiFi Assembly; NEB). For the C-terminal fusions of the SmBit to the human α_1D-AR (pCMV_hmα1DAR-SmBiT) and Alpha1DLight (pCMV_Alpha1DLight-SmBiT, for the luminescence complementation assay) the SmBiT was PCR amplified from the pCMV-nLightG-SmBit plasmid[15] and added using Gibson assembly. The DNA sequence encoding for Alpha1DLight was cloned into a viral vector backbone (#187180; Addgene) under the control of the hSynapsin promotor by PCR amplification and restriction enzyme cloning using *BamHI* and *HindIII* to get the pAAV_hSyn_Alpha1DLight plasmid. The hSynapsin promotor was replaced witha CMV promotor by restriction enzyme cloning using *XbaI* and *PacI* to get the pAAV_CMV_Alpha1DLight plasmid. The rAAVs used in this study were produced by the Viral Vector Facility (VVF) of the University of Zurich. All standard plasmids were amplified in NEB 10-beta Competent *E. coli* and the pAAV plasmids were amplified in.

Cell culture, confocal imaging and quantification

HEK293T cells were seeded into 35 mm glass bottom dishes (D35C4-20-1-N; Cellvis) and transfected at a confluency of 50%–60% with 2 μg of plasmid DNA using PolyFect (301107; QIAGEN) according to the manufacturers protocol. Transiently transfected cells were imaged 24h–48h after transfection.

LLP HEK cells were seeded into standard cell culture media (without additional antibiotics) and transfected 24h later at a confluency of 50–60% with 2 μg of promotor-less attB_α1DAR_SG or attB_Alpha1DLight plasmid DNA using PolyFect according to the manufacturers protocol. Indicator expression was induced 24h after transfection using Doxycycline (2.5 μg/mL) and the selection for recombined cells was started 24h later using Puromycin (1 μg/mL). Cells were passaged ≥3 times prior to using them in experiments. For the dual-color experiments the cell lines were mixed at a 1:1 ratio and seeded into 35 mm glass bottom dishes (D35C4-20-1-N; Cellvis) 24 h prior to the experiment.

Primary embryonic rat cortical neurons were prepared and cultured as described previously[15] After 4 to 6 days *in vitro* (DIV) the neurons were transduced with AAV2/9.hSynapsin.Alpha1DLight at a final titer of $1{*}10^{10}$ GC×mL^{-1} and imaged 14 days later at DIV 18 to 20.

All cells were imaged at RT in imaging buffer (HBSS (14025050; Thermo Fisher Scientific) supplemented with 30 mM HEPES (15630056; Thermo Fisher Scientific)) using a Zeiss LSM 800, with a 40× or 63× oil objective and 488 nm and 561 nm excitation lasers, connected to a computer running the ZEN Microscopy Software (Zeiss). Ligands were dissolved in imaging buffer and manually applied after. A timeseries was recorded to quantify the baseline fluorescence (F_0) and the fluorescence after ligand application (F_t). Fiji (ImageJ) was used to quantify the dynamic range ($(F_t\text{-}F_0)/F_0 = \Delta F/F_0$) upon ligand application. A mean intensity projection of the whole timeseries was used to threshold on the fluorescent plasma membranes of the expressing cells and select ROIs. The ROIs were used to extract the fluorescence intensity values from the timeseries, determine the F_0 and F_t and ultimately determine the $\Delta F/F_0$ of each ROI.

Patch-clamp fluorometry experiments

Alpha1DLight response kinetics upon norepinephrine (NE) application and removal of were measured using patch-clamp fluorometry in combination with fast, piezo-driven ligand perfusion (fast-PCF)[15,46] In brief, HEK293Ttsa cells were seeded on glass coverslips coated with poly-D-lysine (5 μg/mL, Sigma P6407) and laminin (2.7 μg/mL, Sigma L2020) and cultured in Dulbecco's Modified Eagle Medium (DMEM) with 7% (v/v) fetal bovine serum at 37°C and 5% CO_2. Approximately 48 h before the measurements the cells were transiently transfected with 0.4 μg plasmid DNA per milliliter medium using branched polyethylenimine 25,000 (Sigma 408727).

For the measurements, transfected cells were placed in extracellular solution (138 mM NaCl, 1.5 mM KCl, 1.2 mM MgCl2, 2.5 mM CaCl2, 10 mM HEPES, pH 7.3) and outside-out patches were excised at room temperature after establishing cell-attached and whole-cell configurations using a standard patch-clamp setup on an inverted microscope (Leica DMi8) equipped with a micromanipulator (Scientifica Patchstar), patch-clamp amplifier and A/D converter (Molecular Devices Axopatch 200B, Digidata 1550 and pClamp 10.7 software). The patch pipettes were pulled from borosilicate glass (Warner Instruments G150TF-4) and filled with intracellular solution (135 mM K-gluconate, 10 mM NaCl, 2 mM MgCl2, 1 mM EGTA, 10 mM HEPES, pH 7.4) showing a resistance of 6–10 MΩ. The outside-out patches were positioned in front of a θ-barrel perfusion pipette (Warner Instruments TG200-4, outer diameter: 2.0 mm, inner diameter: 1.4 mm, septum: 0.2 mm; tip broken to a diameter of ∼150 nm) that was mounted onto a piezo actuator (Physik Instrumente P842.20)[47] One channel of the perfusion pipette served for perfusion with extracellular solution, the other for NE application. The lateral displacement of the perfusion pipette was triggered by a 6 V ramp in 0.7 ms that was filtered at 1 kHz and amplified with a power supply (Physik Instrumente E505.10), which results in submillisecond switch between the two solutions. NE (1 μM, Sigma 74480) was applied for 2 s in eight successive sweeps with a flow rate of 0.35 mL/min. In addition, the chamber was continuously washed with extracellular solution (flow rate of 3 5 mL/min) using a gravity-driven bath perfusion.

Fluorescence signals from the excised patches were collected through the inverse microscope using a 20× NA 0.40 objective (Leica HC PL FLUOTAR L CORR PH1). Alpha1DLight fluorescence was measured using a 470 nm blue-light LED (Thorlabs M470L3), a 470/40 nm excitation filter, a 495 nm dichroic mirror and a 525/50 nm emission filter (all Chroma). The light intensity at the focal plane was ~0.5 mW/mm^2. Images were acquired with an EMCCD camera (Photometrics Evolve 512delta) and Micro-Manager 2.0b[45] with camera binning 2 × 2 and EM gain 500. Both, the LED and the camera were triggered with TTL pulses programmed in pClamp 10.7 with an LED exposure time of 13 ms and a camera exposure time of 9 ms (camera clearing mode: pre-sequence, trigger mode: strobed). To minimize bleaching during acquisition of the slow off kinetics a split time-based protocol was used (Figure 1), i.e., images were first acquired with a frequency of 40 Hz (5.5 s, starting 1.5 s before the NE application) and later with a frequency of 10 Hz (8.5 s or 14.5 s, starting 2 s after the NE application). Data analysis was performed with ImageJ 1.54f72,[44] Clampfit 11.1 (Molecular Devices), OriginPro 2023 (OriginLab Corporation), and ProFit 7.1 (Quantumsoft). ImageJ was used to determine the patch region and to extract fluorescence intensities. First, mean intensity projections were generated from 25 frames bevor (F_0) and during (Fmax) NE application to calculate a $\Delta F/F_0$ map. Using this map, the patch region was defined and the fluorescence intensity time courses of this region and a neighboring background region were extracted. For background correction, the intensities of the neighboring region were subtracted from the patch region. Next, a baseline value (F_0) was defined from 25 frames before NE application, subtracted from background-corrected intensities to obtain ΔF, and normalized to the baseline intensity to obtain $\Delta F/F_0$. Recordings in which single sweeps had signal-to-noise ratios ≤8 were excluded. Averages of 4 sweeps (all showing less than 12% bleaching between sweeps) were calculated for each recorded patch after performing another baseline correction by subtracting the mean $\Delta F/F_0$ from 25 frames before each NE application from the respective sweep to account for bleaching. This primary analysis and data assessment was performed with Clampfit and OriginPro. The ON and OFF kinetics were analyzed with single exponential fits using ProFit. Fitting of alternative time windows of the slow OFF kinetics suggest that the corresponding OFF time constants might still show a small bias toward slower kinetics when more bleaching occurs, but we did not correct for this effect. Data are given as means ± s.d. with the number of recorded patches (from 2 independent transfections).

Plate-reader-based assays

The fluorescence excitation/emission spectra and the (inhibition) dose-response curves were measured using a Tecan M200 Pro plate-reader.

For the one-photon excitation/emission spectra HEK293T cells transiently expressing Alpha1DLight (pCMV_Alpha1DLight) were used. 48h after transfection the cells were detached using Versene (15040066; ThermoFisher), centrifuged (RT; 3 min; 150 g) and the cell pellet was resuspended in imaging buffer (see above to reach a cell density of $3.33 \cdot 10^6$ cells/mL. The cell suspension was dispensed into a black flat-bottom 96-well plate (150 μL/well) and mixed with 150 μL of imaging buffer with/without NE (20 μM). The plates were incubated at RT for 15 min to equilibrate prior to measuring the excitation spectra at a constant emission wavelength (520 nm, 20 nm bandwidth) or the emission spectra at a constant excitation wavelength (480 nm, 10 nm bandwidth) whilst scanning in 2 nm increments. The autofluorescence was measured using mock-transfected HEK293T cells and subtracted from the signal prior to averaging of biological replicates and normalizing to the maximal fluorescence signal.

For the dose-response curves LLP-HEK cells stably expressing the indicator variants were detached using Versene, resuspended in imaging buffer to $3.33 \cdot 10^6$ cells/mL and dispensed into a black flat-bottom 96-well plate (500′000 cells/well in 150 μL). The ligands were diluted in imaging buffer to reach twice the desired final concentrations and added to the cells (150 μL/well). The plates were incubated at RT for 15 min to equilibrate prior to measuring the fluorescence intensity at 560 nM (20 nM, bandwidth) upon excitation at 488 nm (9 nm bandwidth). The biological replicates were normalized and fitted with four-parameter dose-responses curve to determine the EC_{50}.

For the inhibition dose-response curves LLP-HEK cells stably expressing Alpha1DLight were detached using Versene and resuspended in imaging buffer to 1.66 · 106 cells/mL. NE (L-Norepinephrine-hydrochloride diluted in imaging buffer) was added to the cells at twice the final concentration of 700 nM (≈EC90) and the cells were dispensed into a black flat-bottom 96-well plate (250′000 cells/well in 150 μL). The antagonists (BMY 7378 dihydrochloride, prazosin hydrochloride, risperidone, silodosin, tamsulosin hydrochloride, trazodone hydrochloride and yohimbine hydrochloride) were dissolved in pure DMSO to a stock concentration of 20 mM and diluted in imaging buffer in a serial dilution to reach twice the desired final concentrations and added to the cells (150 μL/well). The final concentration of DMSO in all wells was 0.5% (v/v). The plates were incubated at RT for 20 min to equilibrate prior to measuring the fluorescence intensity at 560 nM (20 nM, bandwidth) upon excitation at 488 nm (9 nm bandwidth). The biological replicates were normalized and fitted with four-parameter dose-responses curve to determine the IC50 and the Ki values were calculated using the Cheng-Prusoff equation:

$$K_i = \frac{IC50}{1+\frac{[NE]}{K_D}}$$

To investigate downstream signaling via G proteins or β-arrestin2 we used a NanoLuc complementation assay as previously described. In brief, we C-terminally fused part of the NanoLuc (SmBiT) to the human α_1D-AR (pCMV_hmα1DAR-SmBiT) or Alpha1DLight (pCMV_Alpha1DLight-SmBiT) and transiently co-expressed either of these constructs in HEK293T cells together with a mini-G protein probe (mini-$G\alpha_s$ (pCMV_LgBiT-miniGs), mini-$G\alpha_{si}$ (pCMV_LgBiT-miniGsi) or mini-$G\alpha_{sq}$ (pCMV_LgBiT-miniGsq))[48] or

β-arrestin2[43] (pNBe3_LgBiT-βarrestin2), N-terminally fused to the complementing part of the NanoLuc (LgBiT). The cells were seeded in 35 mm dishes (250′00 cells/well), transfected 24h later using PolyFect (301107; QIAGEN) according to the manufacturers protocol and harvested 24h later using Versene (15040066, Themo Fisher Scientific). The cells were washed and resuspended in luminescence buffer (Fluorobrite-DMEM (A1896701, Thermo Fisher Scientific) supplemented with 30 mM HEPES (15630056; Thermo Fisher Scientific)) to a density of 0.5 10^6 cells/mL. Of this mixture 200 μL were mixed with 50 μL of 20-fold diluted NanoGlo substrate in LCS buffer (Nano-Glo Live Cell Assay System) and 100 μL/well of this solution was transferred into a white flat bottom 96-well plate (6055290, Revvity) and incubated (45 min; 37°C; 5% CO_2). The luminescence was measured using a Tecan Spark multimode microplate reader (Tecan Group) for 12 min before and 24 min after addition of 20 μL luminescence buffer ± NE (6× concentrated to reach 10 μM final). Luminescence ratios over time were calculated by dividing the signal of the well with the ligand by the signal of the well without ligand. For statistical analysis the mean luminescence ratios after NE addition were compared between the hm α_1D-AR and Alpha1DLight.

Stereotactic surgeries

All surgeries were performed under isoflurane anesthesia (4% induction, 1–2% maintenance) using a stereotaxic frame. Pre-operative analgesia was provided with buprenorphine (0.3 mg/mL, 0.1 mg/kg, subcutaneous, 30 min before surgery) and carprofen (50 mg/mL stock, 5 mg/kg, subcutaneous, 30 min before surgery), and local anesthesia was achieved with a lidocaine hydrochloride (2% w/v, 4 mg/kg, subcutaneous) and bupivacaine (2.5 mg/mL, 2 mg/kg, subcutaneous) mix. Eyes were protected with ophthalmic gel throughout the procedure.

For cerebellar *ex vivo* imaging of Alpha1DLight, a craniotomy was performed above the cerebellar vermis (AP -6.5 mm). AAV8-CMV-Alpha1DLight-WPRE-bGHp(A) (4.4×10^{12} vg/mL; 500 nL) was delivered via manual pressure injection in 100 μm DV increments from 1.7 to 0.5 mm depth. At the end of the procedure, the incision was sealed with tissue adhesive, and mice received post-operative carprofen (10 mg/kg, twice daily for 4 days) and close monitoring during recovery.

For photometry and optogenetic experiments, DBH-iCre mice received unilateral injections of AAV5-hSyn1-dlox-ChrimsonR_tdTomato(rev)-dlox-WPRE-bGHp(A) (5.2×10^{12} vg/mL; 1000 nL) into the locus coeruleus (AP −5.4 mm, ML +1.0 mm, DV −3.8 mm), followed by AAV9-hSyn1-Alpha1DLight (1.8×10^{13} vg/mL; 600 nL) into the lateral hypothalamus (AP −1.4 mm, ML +1.1 mm, DV −5.2 mm). Injections were delivered using pulled glass pipettes and a Nanoject II injector at 13 nL/s. After LHA viral delivery, an optical fiber (400 μm core, NA 0.50) was implanted 0.2 mm above the injection site and secured with dental acrylic. Mice were allowed to recover for 4–5 weeks before photometry and optogenetic experiments.

Acute brain slice preparation and imaging

For preparation of acute brain slices, mice were deeply anesthetized with isoflurane, then decapitated 12–15 days after virus injection. Brains were isolated and transferred into ice-cold cutting solution (87 mM NaCl, 75 mM Sucrose, 2.5 mM KCl, 1.25 mM NaH_2PO_4, 25 mM $NaHCO_3$, 0.5 mM $CaCl_2$, 7 mM $MgCl_2$ and 10 mM D(+)-Glucose bubbled with carbogen (95/5% O_2/CO_2). Sagittal slices (200 μM) comprising the cerebellar vermis were obtained using a vibratome (Leica VT1200), while constantly oxygenated with carbogen. For revitalization, slices were incubated at 34°C for 30 min in oxygenated artificial cerebrospinal fluid (aCSF; 125 mM NaCl, 2.5 mM KCl, 1.25 mM NaH_2PO_4, 26 mM $NaHCO_3$, 2 mM $CaCl_2$, 1 mM $MgSO_4$ and 20 mM D(+)-Glucose (1× hydrate). Slices were kept at RT until recorded. Recordings were performed using the Zeiss LSM 980 with Airyscan 2 from slices maintained in a 32°C microscope chamber and perfused with heated (32°C), oxygenated aCSF with a flow rate of 2.5 mL/min (equally for drug application). For visualization of Alpha1DLight, slices were illuminated with a 488 nm using 3% laser power in Airyscan mode (0.36% in confocal mode). Recordings were performed at a 0.125 sample rate and 0.79 Hz using a 40× objective (LD LCI PlnApo 40×/1.2 W DICIII, Zeiss). Both laser intensity and sample rate were chosen to minimize possible photobleaching. For NE imaging of cerebellar PCs, a 5 min baseline was recorded before infusion of 10 μM NE (L-Norepinephrine-hydrochloride, Sigma-Aldrich, catalog no. 74480) for 1 min, followed by washout with aCSF. To ensure α_{1D}-AR specificity, recordings were initially conducted in the presence of BMY-7378 (α_{1D}-AR antagonist, HelloBio). Slice viability was initially confirmed by infusing 10 μM NE (after 5 min of baseline recording) followed by a subsequent washout. Slices were then treated with the α_{1D}-AR antagonist BMY-7378 dihydrochloride (1 mM BMY-7378 in aCSF) for 1 min, followed by an immediate 1 min co-perfusion of 1 mM BMY-7378 and 10 μM NE. To confirm the indicator's capacity for repeated NE stimulation, a 1 min perfusion of 10 μM NE, followed by a washout and a second application of 10 μM NE was performed. All recordings were maintained for a maximum duration of 40 min. For the analysis of *ex vivo* imaging data, regions of interest (ROIs) were defined within the area expressing the virus using Fiji (ImageJ). For calculation of mean pixel intensity, the ROI manager was used. Calculation of the change in fluorescence ($\Delta F/F_0$ (%)) of ROIs was performed, where $\Delta F/F_0$ (%) is defined as $(F\text{-}F_0)F_0$x100. F_0 was set using the 5 min baseline. For bar charts, peak NE responses were calculated as maximum $\Delta F/F_0$ (%). For experiments involving perfusion of varying NE concentrations the washout (WO) was defined as mean $\Delta F/F_0$ (%) measured during the last 5 min of the recording. For BMY-7378 experiments, and the respective control experiments, the WO was defined as mean $\Delta F/F_0$ (%) recorded during the 5 min immediately preceding the perfusion of BMY-7378 or the second NE application. The number of mice (referred to as N) and slices (referred to as n) together with the performed statistical tests are specified in the correlating figure legends. Statistical analysis was performed using GraphPad Prism 8.2.1.

Experiments with fiber photometry and optogenetics

Fiber photometry recordings were performed using a Doric iFMC6_IE(400–410)_E1(460–490)_F1(500–540)_E2(555–570)_F2(580–680)_S system (Doric Lenses) controlled by Doric Neuroscience Studio (v6.1.2.0). A single low-autofluorescence patch cord

(400 μm core, 0.57 NA; Doric Lenses) was connected to the implanted fiber and served as the common optical path for both photometry and optogenetic stimulation. Alpha1DLight was excited with a 465 nm LED while emitted fluorescence was collected through the same fiber, and a 405 nm LED provided an isosbestic control channel. Both excitation wavelengths were sinusoidally modulated (405 nm: 208 Hz; 465 nm: 572 Hz), demodulated online via lock-in amplification, and low-pass filtered at 12 Hz. Excitation power at the fiber tip was maintained at ~30–60 μW. Mice were habituated to handling, injections, and tethering prior to all fiber photometry experiments.

For the tail-lift paradigm, mice were connected to the photometry patch cord and placed in a neutral recording chamber. The experimenter gently lifted each mouse by the base of the tail so that the body was fully suspended above the cage floor for 60 s. Each session consisted of 5 tail-lift episodes, separated by 180 s inter-trial intervals, while photometry was recorded continuously from baseline through all tail-lift events. To assess the effects of pharmacological manipulations on NE signals, a 5 min baseline photometry recording was acquired prior to intraperitoneal (i.p.) injection, followed by an additional 5 min recording before the first tail lift. Mice received either saline (0.9% NaCl, 10μL/g bodyweight, i.p.) or drug solution. BMY-7378 was diluted in saline and administered at 1 mg/kg bodyweight (for i.p. 10 μL/g). Trazodone (T6154-1G; Sigma Aldrich) was diluted in saline and administered at 10 mg/kg bodyweight (for i.p. 10 μL/g), and Prazosin (hydrochloride; 15023; Cayman Chemicals) was first dissolved in DMSO, then diluted in saline to 1 mg/kg bodyweight for i.p. (10 μL/g).

Optogenetic activation of LC to LHA noradrenergic projections was performed in isoflurane-anesthetized mice (4% induction, 1–2% maintenance) expressing ChrimsonR in LC projections. The same patch cord used for photometry also delivered 638 nm light from a connectorized laser diode module (Doric Lenses), with power at the fiber tip adjusted to ~21 mW based on implant transmission efficiency. Stimulation consisted of four protocols presented in a fixed sequence and separated by 600-s inter-trial intervals: 5 Hz - 1 s, 5 Hz - 5 s, 5 Hz - 10 s, and 20 Hz - 10 s. Laser pulses (50 ms) were generated using a Pulse Pal stimulator[49] Photometry was acquired continuously, and optogenetic triggers were recorded as TTL pulses for post-hoc alignment and analysis. Optogenetically-evoked Alpha1DLight signals were analyzed using the same custom MATLAB pipeline used for tail-lift analysis. Optogenetic stimulation trains were extracted from recorded TTL indices. Each train was aligned to its onset, and peri-stimulus windows were segmented (−5 to +35 s), including a 5-s baseline, the stimulation period, and a post-stimulus response window. For each trial, ΔF/F traces were baseline-corrected using the −5 to 0 s prestimulation period and interpolated onto a common time axis to enable averaging across trials and mice. For each stimulation protocol (5 Hz - 1 s, 5 Hz - 5 s, 5 Hz - 10 s, 20 Hz - 10 s), we computed the average ΔF/F time course per mouse by averaging across all trains of the same protocol. Mouse-level averages were then combined to obtain group mean ± SEM responses. Full-session traces, protocol-aligned averages, and stimulation-sorted heatmaps were generated for visualization. Quantification of response magnitude was performed by extracting, for each trial, the peak ΔF/F during the stimulation window; peak values were averaged per mouse and plotted as group mean ± SEM across protocols. Statistical comparisons across stimulation protocols were performed using repeated-measures ANOVA, Greenhouse–Geisser corrected with Tukey's multiple comparisons test.

Tail-lift photometry data were analyzed using a custom MATLAB script. For each mouse, the demodulated 465 nm (Alpha1DLight) and 405 nm (isosbestic) channels were loaded, cleaned for missing values, low-pass filtered at 3 Hz, and truncated to a common length. Motion artifacts and shared noise were removed by regressing the 465 nm signal onto the 405 nm signal, and the fitted baseline (F_0) derived from the 405 nm channel was used to compute percent ΔF/F according to $\Delta F/F_0 = (F(t) - F_0)/F_0$, where $F(t)$ is the 465 nm signal. Tail-lift events were identified using recorded event indices and used to extract peri-event windows from −15 to +170 s relative to lift onset (15 s baseline, 60 s lift, 110 s post-lift). For each trial, ΔF/F traces were baseline-corrected using the −15 to 0 s prestimulus period and interpolated onto a common time axis to allow averaging across events and mice. Four response metrics were quantified per trial: peak amplitude, defined as the maximum ΔF/F value within the 0–60 (+5 extra) s lift period; latency to peak, defined as the time from lift onset to this maximum value; area under the curve (AUC), computed as the trapezoidal integral of the ΔF/F trace from 0 to 60 s; and the decay constant τ, obtained by fitting a single-exponential function to the post-peak portion of the trace from peak time to the end of the 0–170 s window. Metrics were averaged across trials and mice, and group means ± SEM were reported. Full-session traces, event-aligned averages, and event-sorted heatmaps were generated for visualization.

For fiber photometry experiment during recovery from anesthesia, recordings were acquired during isoflurane (ISO) anesthesia and aligned to anesthetic offset (ISO-OFF). Demodulated 465 nm and 405 nm signals were processed using a custom MATLAB pipeline, low-pass filtered at 3 Hz, and corrected for motion artifacts by linear regression of the 465 nm channel onto the 405 nm channel. Percent fluorescence change (ΔF/F) were computed using a −10 to 0 s baseline preceding ISO-OFF. Full-session traces were aligned to ISO-OFF, interpolated onto a common time base, and averaged across mice (mean ± SEM). Latency to NE signal rise was defined for each mouse as the first time point after ISO-OFF at which ΔF/F exceeded 2 standard deviations above baseline for at least 2 s. Mean ΔF/F before ISO-OFF (−10 to 0 s) was compared to a post ISO-OFF window of equal duration (0 to +10 s) anchored to the individually detected latency using a two-tailed paired *t* test.

Histology

Mice were deeply anesthetized with ketamine/xylazine (300/30 mg/kg, respectively, i.p) and transcardially perfused with 1× PBS (pH 7.4), followed by fixation with ice-cold formaldehyde (FA at 4% in 1× PBS, pH 7.4). Brains were dissected and post-fixed in ice-cold 4% FA for 1h before cryoprotection in 30% sucrose in 1× PBS at 4°C. 30 μM thick sagittal brain slices were obtained using a cryostat (Leica CM3050 S). Slices were mounted with Mowiol plus DAPCO (Carl Roth GmbH + Co KG). Images were acquired using a confocal

microscope (Zeiss LSM 980 with Airyscan 2) interfaced to a computer running the ZEN Microscopy Software (Zeiss). AAV-CMV-Alpha1DLight expression was visualized and confirmed using a blue laser (488 nm). Further processing of images was performed with Fiji (ImageJ).

QUANTIFICATION AND STATISTICAL ANALYSIS

Statistical analysis

Data are presented as mean ± SEM. Statistical analyses were performed using parametric or non-parametric tests as appropriate, based on the distribution and experimental design of each dataset. Analyses were conducted using GraphPad Prism 10 or custom-made Python scripts. The specific statistical tests, sample sizes, and exact p-values are reported in the corresponding figure legends. For comparisons between two groups, two-tailed paired or unpaired t-tests were used as appropriate. For comparisons involving three or more groups, one-way or repeated-measures ANOVA was used where applicable, with Greenhouse–Geisser correction applied when sphericity was violated, followed by Tukey's multiple-comparisons test. Statistical significance was defined as: $^*p < 0.05$, $^{**}p < 0.01$, $^{***}p < 0.001$, or $^{****}p \leq 0.0001$.

Cell Reports Methods, Volume *6*

Supplemental information

Subtype-resolved norepinephrine imaging with an engineered α_1D-adrenergic receptor indicator

Valentin Lu Rohner, Zacharoula Kagiampaki, Pauline Bohne, Antonia Renate Klein, Latife Sönmez, Laura Moreno Wasielewski, Musadiq A. Bhat, Andreas Reiner, Melanie D. Mark, and Tommaso Patriarchi

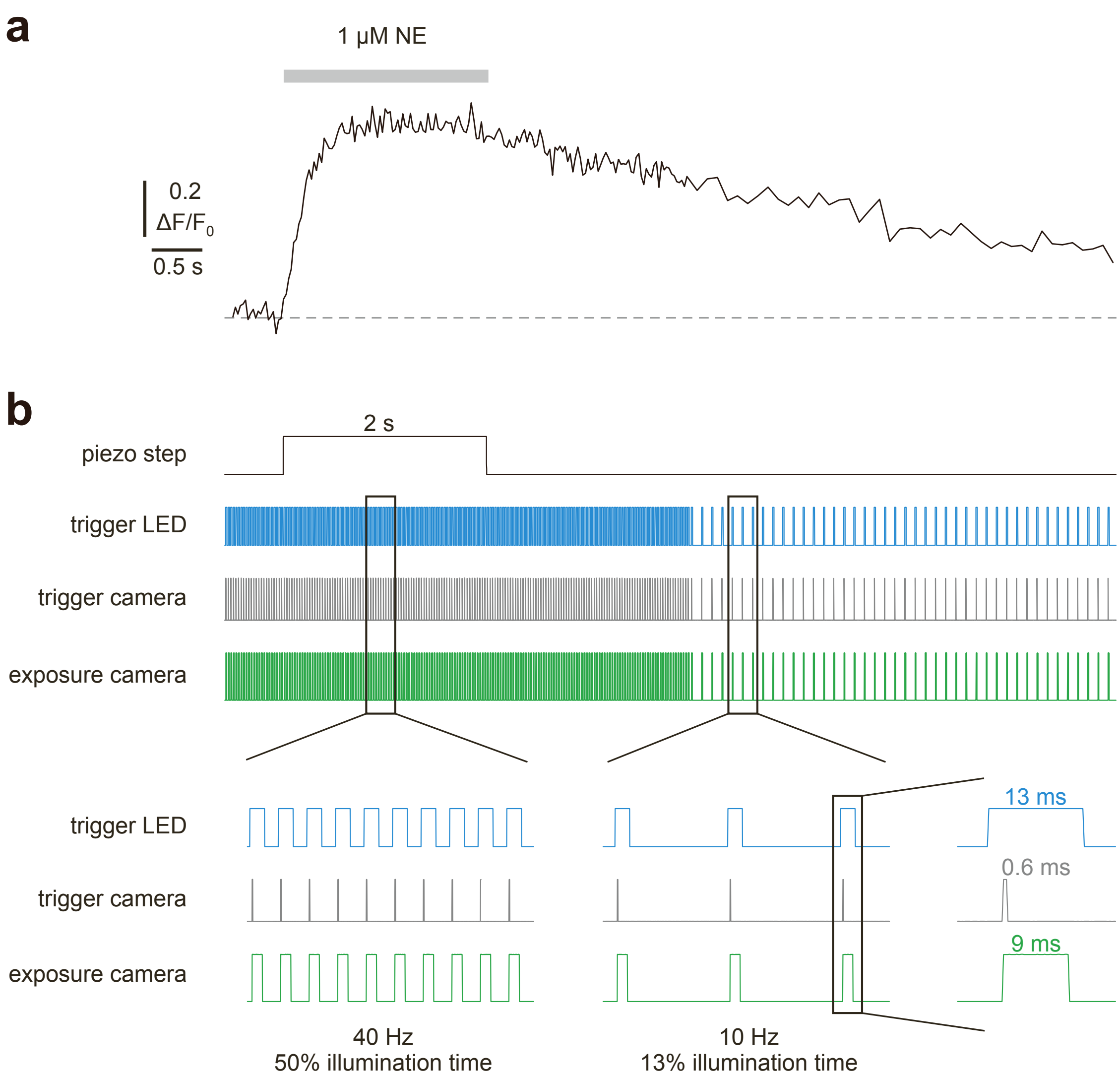

Supplementary Figure 1 | Patch-clamp fluorometry measurements with a split time-based protocol.

a, $\Delta F/F_0$ trace of Alpha1DLight (shortened from **Fig. 1h**). **b,** Trigger signals for the piezo element (solution exchange), 470 nm LED (illumination), camera acquisition (exposure start) and output signal denoting the actual camera exposure. As indicated, 2 s after the 2 s NE application the illumination and sampling episodes were reduced from 40 Hz to 10 Hz to achieve extended measurements times (14 - 20 s per sweep) with reduced photobleaching (illumination time reduced from 50% to 13%).

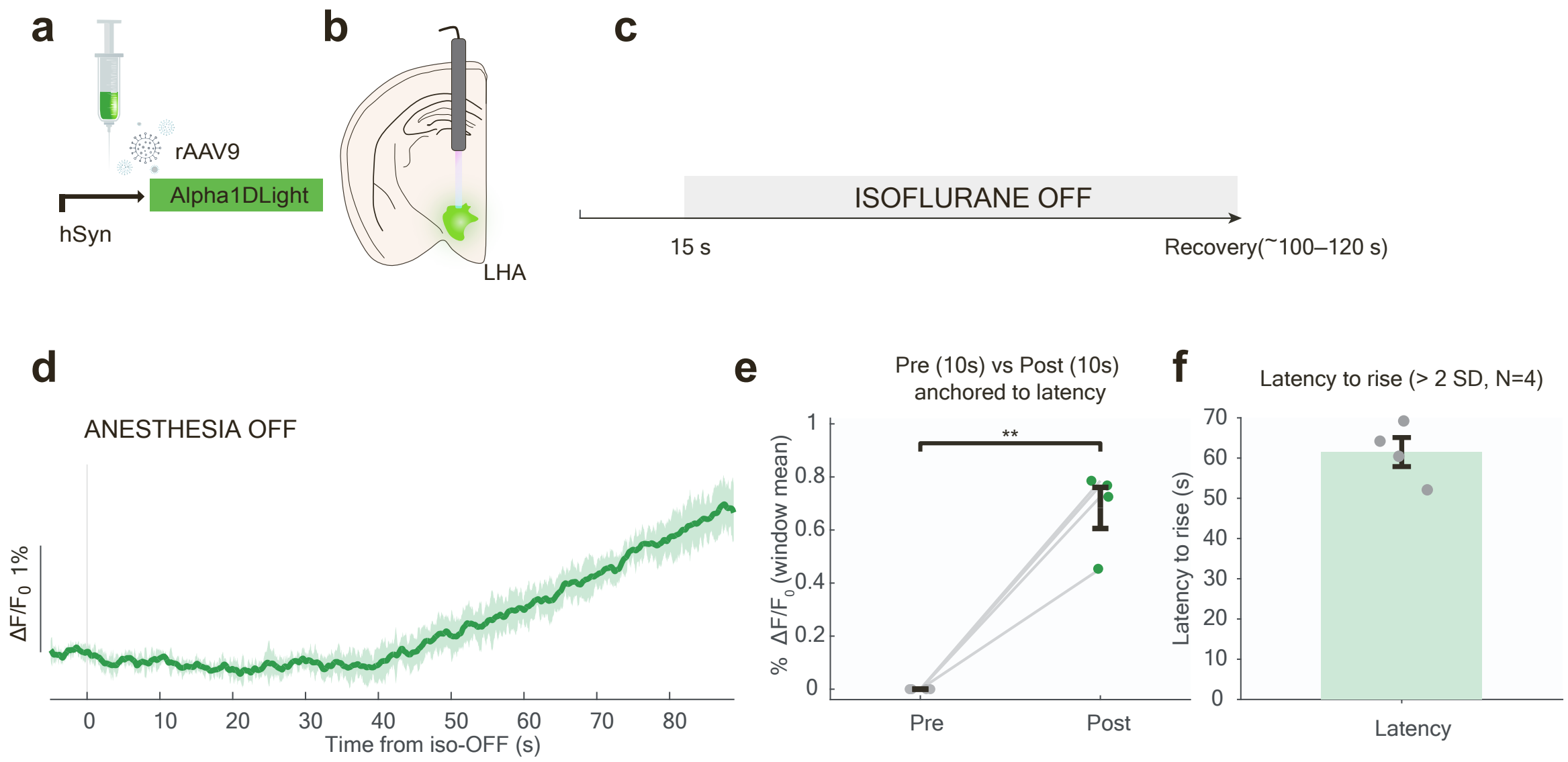

Supplementary Figure 2 | Alpha1DLight reports gradual NE increase upon recovery from isoflurane anesthesia

a-c, Schematic of Alpha1DLight expression in the lateral hypothalamus (LHA) and photometry recordings during isoflurane anesthesia till recovery. **d**, Averaged ΔF/F trace aligned to isoflurane offset (ISO-OFF), showing a gradual tonic increase in NE signal during recovery from anesthesia. **e**, Quantification of mean ΔF/F before isoflurane offset (−10 to 0 s relative to ISO-OFF) and after the onset of NE signal rise (0 to +10 s, relative to the detected latency for each mouse). NE levels were significantly increased following signal onset (two-tailed paired t-test, $t(3) = 8.82$, $p = 0.0031$; N = 4 mice). **f**, Latency to NE signal rise following ISO-OFF, defined as the time to exceed 2 standard deviations above baseline (N = 4 mice). Data are shown as mean ± SEM.
